# Board game-based education for HIV prevention among rural older adults: a quasi-experimental study

**DOI:** 10.3389/fpubh.2026.1834731

**Published:** 2026-10-01

**Authors:** Juan Guo, Qi Yang, Lihong Zhou

**Affiliations:** 1 Hunan Normal University Health Science Center, Hunan Normal University, Changsha, China; 2 Changde Girls’ Foreign Language School, Changde, China

**Keywords:** board games, health education, HIV, older adults, rural areas

## Abstract

**Background and aim:**

Population aging has increased the burden of HIV infection among older adults, particularly in rural areas where access to effective health education remains limited. Traditional educational approaches often fail to engage this population. Board game-based health education has emerged as a promising strategy to enhance learning engagement and knowledge retention.

**Method:**

A quasi-experimental study was conducted among 100 rural older adults in Hunan Province, China. Participants were allocated to either an intervention group (*n* = 50) receiving a board game-based HIV education program or a control group (*n* = 50) receiving conventional lecture-based education. Both groups participated in four weekly sessions covering identical HIV-related content. Outcomes, including HIV-related knowledge, attitudes, prevention behaviors, and condom use self-efficacy, were assessed before and after the intervention.

**Results:**

A total of 97 participants completed the study (intervention group: *n* = 48; control group: *n* = 49). No significant differences in baseline characteristics were observed between groups (*p* > 0.05). Following the intervention, both groups showed significant improvements in all outcomes (*p* < 0.05). However, the intervention group demonstrated significantly greater improvements in HIV-related knowledge, attitudes, behaviors, and skills compared with the control group (*p* < 0.05). Condom use self-efficacy was also significantly higher in the intervention group after the intervention (*p* < 0.05).

**Conclusion:**

Board game-based health education may provide a feasible, low-cost, and culturally adaptable approach for HIV prevention among rural older adults in resource-limited settings. By combining interactive learning, peer discussion, and repeated practice, this approach has potential to complement conventional community health education and support age-appropriate HIV prevention efforts. Future studies with larger samples and longer follow-up periods are needed to confirm the sustainability of the intervention effects and their translation into real-world preventive behaviors.

## 1 Introduction

With the acceleration of global population aging, HIV infection among older adults has increasingly become a prominent public health challenge. The World Health Organization estimates that the proportion of the global population aged 60 years and older will rise from 12% in 2015 to 22% by 2050 (1). In parallel, the population of older adults living with HIV is also substantial. According to data from the Joint United Nations Programme on HIV/AIDS (UNAIDS), approximately 39.9 million people were living with HIV worldwide in 2022, including about 7.5 million people aged 60 years and older (2). The convergence of population aging and the increasing HIV burden in later life indicates that HIV prevention, diagnosis, treatment, and care among older adults require growing attention. Although the magnitude of this trend varies across countries and regions, HIV infection and prevention among older adults have also become important concerns in low-and middle-income settings experiencing rapid population aging (3, 4).

In China, this trend is particularly pronounced. The proportion of older adults among reported HIV/AIDS cases increased sharply from 6.3% in 2005 to 44% in 2020 (5, 6). Consequently, older adults have become a key population for HIV prevention and control, urgently requiring public health attention. It should be noted, however, that the definition of “older adults” varies across research fields and countries. In HIV research, given that HIV infection accelerates immune aging, individuals aged ≥50 years are typically defined as “older adults” (7). By contrast, China’s Law on the Protection of the Rights and Interests of the Older adult(s) legally defines older adults as citizens aged 60 years and above, a standard widely adopted in epidemiological studies of China’s aging population (8, 9). To maintain consistency with the characteristics of the target population and the policy context, this study adopted the Chinese national demographic standard defining older adults as ≥60 years. Accordingly, all mentions of “older adults” in this paper refer specifically to individuals aged 60 years and above.

Compared with their urban counterparts, rural older adults are more likely to be neglected in HIV prevention and control efforts. Due to lower levels of education, limited economic means, and restricted access to health information, rural older adults commonly face insufficient HIV-related knowledge, low risk perception, and low condom use rates (6, 10). Moreover, HIV-related stigma remains pervasive in rural communities, which not only exacerbates fear and avoidance of the disease but also reduces willingness to seek testing and prevention services (11). In some rural areas, access to HIV testing and counseling services is also limited. Even when engaging in high-risk behaviors, older adults may miss critical opportunities for early detection due to limited awareness of the importance of proactive testing (12, 13). Similar structural barriers, including limited educational resources, poor health information dissemination, restricted access to healthcare services, and deeply rooted social stigma, have also been reported in resource-limited rural settings globally (14–16), suggesting that research on HIV prevention among rural older adults has relevance beyond the local context.

Health education is a fundamental strategy for reducing high-risk behaviors and promoting preventive measures. However, current HIV education programs primarily target traditional high-risk groups, such as adolescents and men who have sex with men, while the needs of rural older adults remain largely unmet (17, 18). Moreover, existing educational approaches have limited effectiveness in this population. Traditional offline methods, such as lectures and billboards, often rely on one-way information delivery and may not sufficiently engage older adults with age-related declines in perception, limited health literacy, or low motivation for active learning (19). Digital platform-based health education is constrained by the digital divide, making it difficult to effectively reach rural older adults (20). These barriers indicate that HIV health education for rural older adults should move beyond passive information transmission. Educational strategies should actively engage participants, present HIV-related risks in familiar life contexts, provide opportunities for peer discussion of sensitive topics, and reinforce preventive knowledge and skills through repeated practice and feedback.

Board game–based health education offers a promising alternative for addressing these needs. Board games are structured, rule-driven activities in which players interact within defined systems, such as Ludo or Snakes and Ladders. When integrated with health education, they can transform abstract knowledge into engaging, interactive experiences through scenario-based learning. This format can operationalize the educational mechanisms described above by encouraging active participation, supporting peer interaction, and providing repeated opportunities for feedback and reinforcement. Previous studies have demonstrated the effectiveness of board game-based interventions across various domains, including infectious disease prevention and chronic disease self-management (21–24), indicating their broad applicability in health promotion. For rural older adults, board games may be particularly suitable because they do not require electronic devices or internet access, provide face-to-face interaction, have low technological requirements, and create a relatively relaxed atmosphere for discussing sensitive health topics such as HIV prevention. Nevertheless, empirical evidence remains limited regarding board game-based HIV health education interventions specifically targeting rural older adults, particularly in relation to improving HIV-related knowledge, reducing stigma, and promoting preventive behaviors.

From a theoretical perspective, the effectiveness of board game-based health education can be explained by multiple behavioral science theories. Social Cognitive Theory posits that individuals can develop new behavioral cognitions and self-efficacy through observational learning, scenario-based simulation, and repeated practice. The peer interaction, role modeling, and immediate feedback mechanisms inherent in board games help participants strengthen risk perception and build behavioral confidence during the interactive process (25). The Health Belief Model further suggests that whether an individual adopts a health behavior depends on their perceptions of disease severity, susceptibility, benefits of the recommended action, and barriers to performing it (26). Through contextualized questions and behavioral tasks, the board game in this study helped rural older adults more intuitively understand HIV infection risks and the importance of preventive behaviors, thereby enhancing their willingness to take preventive action. In addition, game-based learning and educational game design frameworks suggest that structured rules, clear goals, challenges, collaboration, competition, and feedback can enhance learning motivation, engagement, and knowledge internalization (27). Consequently, compared with traditional lectures, board game–based health education may reduce psychological resistance to learning and increase sustained participation through its enjoyable and interactive design.

Based on this background, this study designed a board game-based HIV health education intervention informed by behavior change theories and conducted a quasi-experimental study among rural older adults in Hunan Province, China. The study aimed to evaluate the effectiveness of this intervention, compared with conventional lecture-based health education, in improving HIV-related knowledge, attitudes, preventive behaviors, and condom use self-efficacy. We hypothesized that the board game-based health education intervention would produce greater improvements than conventional lecture-based education.

## 2 Materials and methods

This quasi-experimental study aimed to evaluate the effectiveness of a board game-based health education intervention on HIV-related knowledge, attitudes, and behaviors among rural older adults in Yueyang City, Hunan Province, China. The study was conducted between June and September 2025. Administrative villages were used as the unit of allocation. Two villages were randomly selected and assigned to either the intervention group or the control group using a simple randomization method. The intervention group received board game-based health education, whereas the control group received conventional lecture-based HIV health education.

### 2.1 Sample size

Sample size estimation was performed based on the HIV knowledge awareness rate as the primary outcome measure. According to pilot study results, the expected post-intervention knowledge awareness rate was 92% in the intervention group and 70% in the control group, with a corresponding effect size of Cohen’s h ≈ 0.60. A two-sided significance level of *ɑ* = 0.05 and a power of 80% (1-*β* = 0.80) were set. Sample size calculation was performed using PASS 25.0 software, yielding a required sample size of 45 participants per group. Accounting for a 10% attrition rate, the final sample size was set at 50 participants per group, with a total of 100 participants enrolled in the study.

### 2.2 Participant recruitment

A registry of residents aged ≥60 years was obtained from village committees. With the assistance of local village physicians, eligible participants were approached through door-to-door visits. Trained research staff provided detailed explanations of the study objectives, procedures, potential benefits, and risks. Participants were informed of the voluntary nature of participation and their right to withdraw at any time without penalty. Written informed consent was obtained prior to enrollment.

No gender restrictions were imposed during the recruitment process, and eligible male and female rural older adults were eligible for inclusion. The recruitment strategy aimed to ensure that the gender distribution of the sample aligned with the actual demographics of the rural older adult population in the study area. According to local village registration data, males accounted for approximately 45% and females approximately 55% of residents aged 60 years and above. The gender composition of the final sample is detailed in the Results section (Table 1).

**Table 1:** Participant demographics (*n* = 97).

Characteristics	Intervention group (*n* = 48)	Control group (*n* = 49)	*t/χ* ^2^	*p*
Gender			0.257	0.612
Male	25 (52.1)	23 (46.9)		
Female	23 (47.9)	26 (53.1)		
Age (years), mean (SD)	65.90 ± 2.845	66.33 ± 2.478	−0.795	0.428
Marriage, *n* (%)			1.013	0.87
Unmarried	1 (2.1)	1 (2.0)		
Married	31 (64.6)	33 (67.3)		
Cohabitation	4 (8.3)	2 (4.1)		
Widowed	12 (25.0)	13 (26.5)		
Occupation, *n* (%)			3.302	0.685
Farming	21 (43.8)	19 (38.8)		
Migrant Worker	2 (4.2)	5 (10.2)		
Self-Employed	3 (6.2)	5 (10.2)		
Retired	1 (2.1)	3 (6.1)		
No job	18 (37.5)	15 (30.6)		
Other	3 (6.2)	2 (4.1)		
Monthly income			1.665	0.676
<500	36 (75.0)	33 (67.3)		
500–1,000	9 (18.8)	13 (26.5)		
1,001–1,500	1 (2.1)	2 (4.1)		
>1,500	2 (4.2)	1 (2.0)		
Education, *n* (%)			1.121	0.854
Illiterate	11 (22.9)	10 (20.4)		
Primary school	32 (66.7)	31 (63.3)		
Junior high school	4 (8.3)	7 (14.3)		
Academic	1 (2.1)	1 (2.0)		
Inhabiting information, *n* (%)			0.335	0.563
Solitary	5 (10.4)	7 (14.3)		
Living with spouse/children/grandchildren, etc.	43 (89.6)	42 (85.7)		
Have you heard of AIDS?			0.384	0.536
Yes	40 (83.3)	43 (87.8)		
No	8(16.7)	6 (12.2)		
Have you received AIDS health education?			–	–
Yes	48 (100.0)	49 (100.0)		
No	0 (0.0)	0 (0.0)		
Access to AIDS information (multiple choices available), *n* (%)			2.909	0.781
TV/broadcast	29 (60.4)	24 (49.0)		
Internet	14 (29.2)	15 (30.6)		
Newspaper	0 (0.0)	1 (2.0)		
Community and hospital publicity	1 (2.1)	3 (6.1)		
Children/relatives/friends inform	2 (4.2)	3 (6.1)		
Other	2 (4.2)	3 (6.1)		

### 2.3 Inclusion/exclusion

Inclusion criteria: (1) aged 60 years or older; (2) registered rural residents; (3) residence in the rural community for at least 6 months; (4) able to communicate effectively, understand simple oral instructions in Chinese, and judged by trained investigators to have no obvious cognitive impairment that would prevent participation; (5) able to read simple written Chinese or able to participate in the board game with facilitator- or peer-assisted reading; (6) provision of written informed consent.

Exclusion criteria: (1) severe visual, hearing, or speech impairments that may hinder participation; (2)terminal illness or severe acute or chronic conditions that would prevent participation in a 30-45-min group-based educational session or completion of the study questionnaire; (3) prior participation in HIV/AIDS-related educational interventions.

Limited literacy was not considered an exclusion criterion. During gameplay, if a participant was unable to read the card text independently, the facilitator or a peer read the card aloud verbatim without providing additional explanations or hints before the participant responded. After the response, the facilitator provided feedback and explanations according to the standardized reference answers. All facilitators received standardized training before the intervention to ensure consistency in card reading, feedback, and explanation.

### 2.4 Randomization and blinding

This study used administrative villages as the unit of randomization. A simple randomization method was employed to assign the two villages to either the intervention group or the control group. The specific procedure was as follows: Before study initiation, an independent researcher who was not involved in intervention implementation or data collection was responsible for the randomization process. This researcher determined the group assignment of the two villages by coin toss (heads: intervention group; tails: control group) and recorded and preserved the randomization results. To ensure allocation concealment, the randomization results were sealed in sequentially numbered, opaque, sealed envelopes. Each envelope was labeled with a unique number on the outside and contained the corresponding group assignment information. All envelopes were kept by the independent researcher. After completion of all baseline assessments, the envelopes were opened in sequential order to reveal the group assignment for each village. Due to the nature of the interventions (board game vs. health lecture), blinding of participants and intervention facilitators was not feasible. However, the data analysts remained blinded to group assignment during the statistical analysis phase to reduce analytical bias. To minimize contamination between study groups, participants were asked to refrain from discussing intervention-related content across villages during the study period.

### 2.5 Pilot study

A pilot study was conducted from March to April 2025 prior to the formal trial, involving 12 rural older adults aged 60–75 years from villages not included in the main study. The pilot objectives were: (1) to evaluate the acceptability and comprehensibility of the board game design; (2) to assess the appropriateness of the language, question difficulty, and visual design of the educational cards; (3) to determine the optimal single-session duration and group size; and (4) to test the feasibility of the study procedures.

Based on pilot feedback, the following adjustments were made: (a) the font size on the game board and cards was enlarged to accommodate age-related visual decline; (b) three question cards with overly complex wording were revised; (c) the single-session duration was shortened from 45–60 min to 30–45 min to align with participants’ attention spans; and (d) group size was limited to 2–4 participants to ensure adequate interaction and facilitator support. The pilot study confirmed the feasibility and acceptability of the board game intervention among the target population.

### 2.6 Study timeline

This study was conducted from June to September 2025, adhering to the following timeline:

Week 0 (Baseline Assessment, T0): Participants completed a baseline questionnaire to evaluate sociodemographic characteristics, HIV-related knowledge, attitudes, behaviors, skills, and condom use self-efficacy.
Week 1–4 (Intervention Period): The intervention group received board game-based education (once weekly, lasting 30–45 min per session), while the control group received lecture-based education with the same frequency and duration.
Week 5 (Post-Intervention Assessment, T1): Assessments were completed within 1 week after the intervention concluded. All evaluations were conducted by researchers who remained blinded to group allocation.

### 2.7 Intervention

A total of 100 eligible participants were enrolled, with 50 assigned to the intervention group and 50 to the control group. The intervention group received health education through an HIV-themed board game, while the control group attended an HIV-themed lecture delivered by local healthcare workers.

#### 2.7.1 Intervention group

##### 2.7.1.1 Intervention format

A total of 50 participants were assigned to the intervention group. After allocation to the intervention group, participants were sequentially assigned to fixed small groups of 2–4 participants according to enrollment order. This resulted in 13 groups: 12 groups of four participants and one group of two participants. Small-group formation was used only to deliver the intervention and was not part of the group allocation process. Group membership remained unchanged throughout the four-week intervention period, except in cases of participant withdrawal or loss to follow-up. Each group participated in one HIV-themed board game session per week, with each session lasting 30–45 min. Therefore, 13 small-group board game sessions were delivered each week, resulting in a total of 52 small-group sessions over the four-week intervention period. Three trained facilitators were responsible for delivering the intervention. Each facilitator was assigned 4–5 fixed groups and conducted the sessions for these groups sequentially according to a prearranged schedule. Each small-group session was led by one facilitator throughout the session. The facilitator was responsible for explaining the game rules, maintaining the flow of gameplay, supervising card reading and responses, providing immediate feedback and explanations based on standardized reference answers, and ensuring consistency of intervention delivery across groups. Detailed weekly intervention plans and procedures for the board game are provided in Tables 2, 3.

**Table 2:** Weekly intervention plan for the board game.

Weeks	Game theme	Core objective	Card distribution	Duration per card
Week 1	Fundamentals of HIV- related knowledge	Master fundamental concepts, transmission routes, and prevention principles of HIV	14 Knowledge Cards	Average Time per Round: 2.1 min: ①Roll the die and move: 15 s ②Draw a card and read aloud: 20 s ③Thinking time: 30 s ④Answer: 20 s ⑤Judge’s decision and explanation: 30 s ⑥Positive reinforcement: 10 s
Week 2	HIV- related stigma and discrimination	Reduce discrimination and fear while fostering empathy	13 Attitude Cards + 1 Knowledge Review Card	
Week 3	Behavioral skills practice	Master key HIV- prevention behaviors in daily life	12 Behavior Cards + 1 Knowledge Review Card + 1 Attitude Review Card	
Week 4	Integrated Application and Reinforcement	Reinforce learned knowledge, attitudes, behaviors, and skills	10 Knowledge Cards (8 New+ 2 Review) + 1 Attitude Card + 2 Behavioral Cards	

**Table 3:** Intervention process of the board game.

Phase	Duration	Content	Detailed steps
① Introduction or review	5 Minutes	Content quiz and knowledge check	In Week 1, introduce the game rules; from Weeks 2 to 4, reinforce knowledge through quick quizzes.
② Theme introduction	3 Minutes	Introducing this week’s theme	Story-Hook: Start with ‘The Day Zoe Learned About HIV’. In the story, the character mistakes mosquito bites for a transmission route and then corrects her understanding with a doctor’s help.
③ Board game activity	30 Minutes	Thematic game interaction	Roll the die → Move your token → Pick a card → Answer (e.g., True or False: HIV can spread through hugging?)
④ Summary & reinforcement	7 Minutes	Review & summary	Review key points (3 min) + Your turn to share (2 min) + Answer questions (2 min)

##### 2.7.1.2 Game rules

Participants were divided into two teams per group, each selecting a game piece. Turn order was determined by dice rolling. Teams alternately drew question cards and responded within 30 s. For participants with limited literacy, facilitators or peers read the card text aloud verbatim, and participants responded orally. Facilitators provided feedback only after the participant had answered, using standardized reference answers. Correct answers allowed advancement on the board, while incorrect answers resulted in no movement. Facilitators adjudicated disputes and provided standardized explanations.

The winning criteria were as follows: (1) being the first to reach the finish line; (2) advancing the furthest when all cards were used; (3) answering a tie-breaker question correctly if necessary.

Peer discussion was incorporated into the gameplay at several points. Before responding to card questions, team members were allowed to briefly discuss their answers within the group. For Attitude Cards and Behavior Cards involving misconceptions, stigma, risk perception, or preventive behaviors, facilitators provided standardized feedback after the team response and then guided a brief discussion to help participants reflect on the issue. During the summary and reinforcement phase, the “Your turn to share” activity further encouraged participants to voluntarily share one new point they had learned, one misconception that had been corrected, or one HIV prevention behavior they considered feasible in daily life. To ensure consistency across groups, facilitators used standardized prompts, such as “What is one thing you learned today?”, “What is one misunderstanding that was clarified?”, and “What preventive action do you think is feasible in daily life?” Given the sensitive nature of HIV-related topics, facilitators reminded participants that they could discuss general opinions or hypothetical situations and were not required to disclose personal experiences, sexual history, or HIV testing status. Facilitators emphasized respect, confidentiality, and non-judgmental discussion, corrected misinformation when necessary, and guided the discussion toward HIV prevention knowledge and feasible preventive behaviors.

##### 2.7.1.3 Game materials

The game consisted of a board (Figure 1), two pieces, one die, and 53 educational cards categorized as knowledge, attitude, and behavior cards (Figure 2). The educational content was developed based on relevant empirical literature on HIV prevention education and was organized according to the Knowledge-Belief-Behavior (KBB) model (28, 29). Specifically, knowledge cards addressed HIV transmission and clinical features; attitude cards focused on risk perception and social attitudes; and behavior cards targeted preventive practices and testing intentions. Some Attitude Cards and Behavior Cards used scenario-based wording to present HIV-related risk situations, attitude reflection, and preventive behaviors in everyday contexts; however, these cards remained classified as Attitude Cards or Behavior Cards rather than as a separate “scenario card” category. The story-hook or scenario prompts described in Table 3 were used as part of the session procedure and were not a separate category of educational cards.

**Figure 1:**
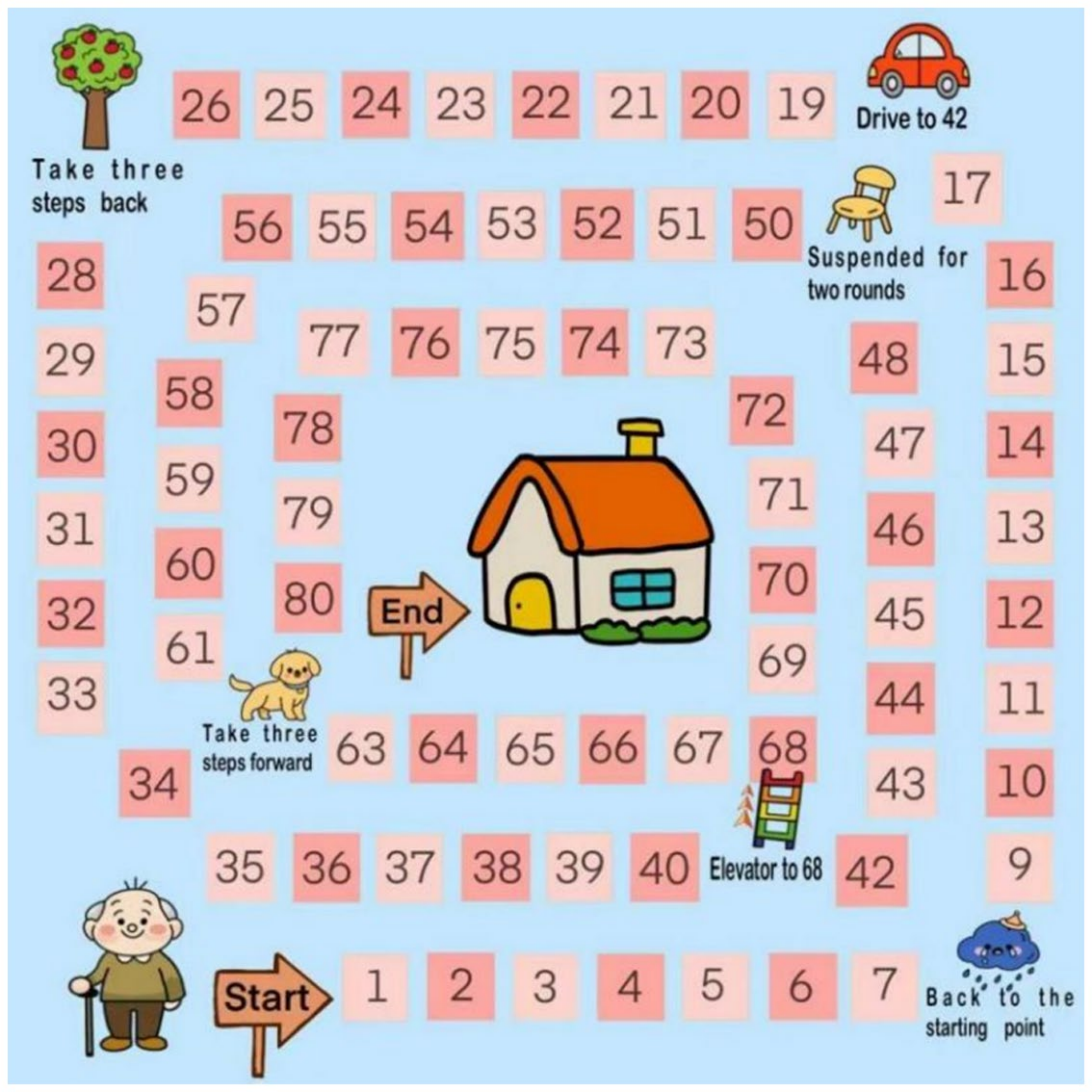
Board game diagram.

**Figure 2:**
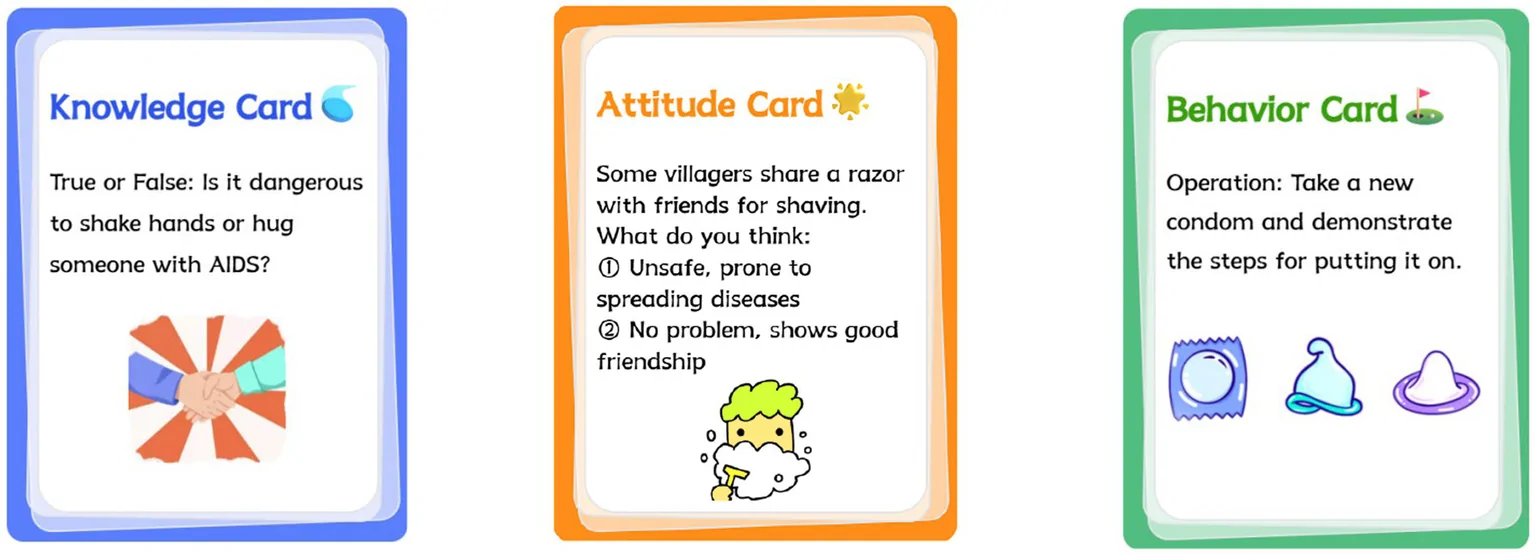
Sample educational cards.

##### 2.7.1.4 Theoretical framework of the intervention

The board game intervention in this study was designed based on Social Cognitive Theory, the Health Belief Model, and game-based learning frameworks. Social Cognitive Theory emphasizes the role of observational learning, behavioral modeling, and self-efficacy in behavior change. Accordingly, this study used group interaction, peer discussion, and behavioral practice to enhance participants’ confidence in and mastery of HIV preventive behaviors. The Health Belief Model posits that an individual’s adoption of health behaviors is closely related to their perceptions of disease risk, severity, and the benefits of taking action. In this study, Attitude Cards and Behavior Cards with scenario-based content presented real-life HIV-related risk situations, attitude-related reflection, and preventive actions, helping participants increase their awareness of HIV risk and strengthen their motivation for active prevention. Game-based learning frameworks highlight the role of competition, rewards, cooperation, and immediate feedback in enhancing learning motivation. Therefore, this study incorporated board progression, team competition, collaborative discussion, and immediate feedback mechanisms into the board game to improve engagement, learning interest, and knowledge retention.

##### 2.7.1.5 Cognitive adaptation strategies for rural older adults participants

To align with the cognitive and sensory profiles of rural older adult populations, the following adaptations were implemented: (a) The game board used high - contrast color schemes. For card text, an 18-point bold font was used. For key information on the game board, a minimum 28-point bold font was employed. Graphics were combined with text to aid comprehension for participants with limited literacy. (b) All written materials were presented in simplified Chinese with concise sentence structures and everyday vocabulary, and were supplemented by local Hunan dialect expressions where appropriate to enhance understanding among participants. (c) Based on pilot testing, to prevent information overload, each card presented only one concept, each game session was limited to 30–45 min, and facilitators repeated key information as needed. (d) Three facilitators underwent a 3-h training program which focused on communication strategies for older adult populations, including speaking at a reduced pace, using plain language, providing positive reinforcement, and addressing potential confusion or frustration. (e) Before the first session, a 5-min familiarization session was held, in which facilitators explained the rules through simplified demonstrations and allowed participants to practice dice-rolling and card-reading without time pressure.

#### 2.7.2 Control group

The control group received conventional thematic health lectures, which were conducted at the village service center. The core content of the lectures (covering HIV transmission routes, clinical manifestations, treatment, policies and regulations, and preventive measures, etc.) was completely consistent with the knowledge points of the board game used in the experimental group, ensuring the homogeneity of the intervention content. A uniformly trained researcher served as the lecturer. The lectures were delivered in the form of PPT presentations combined with the distribution of promotional leaflets, without the use of board game-based or interactive gameplay activities. The lectures were held once a week, each lasting 30 to 45 min, for a total of 4 times. Table 4 presents the thematic lecture content.

**Table 4:** The plan for the thematic lectures.

Weeks	Core theme	Key knowledge points	Time allocation
Week 1	Basic knowledge of HIV	① Basic concepts of AIDS. ② Transmission routes and non-transmission routes ③Prevention principles ④ Current situation and hazards.	30-min PPT explanation + 10-min Q&A + 5- min material distribution.
Week 2	Correct view on HIV	① HIV is not far from rural older adults. ② Hazards of discrimination. ③Scientific treatment of infected individuals. ④ Cultivation of empathy. ⑤ Importance of family support.	30-min PPT explanation + 10-min case discussion + 5-min Q&A.
Week 3	Prevention behaviors and skills	① High-risk behaviors in daily life. ② Correct understanding of condoms. ③ Correct usage methods of condoms. ④ Ways to obtain condoms. ⑤ Safe medical treatment and avoidance of blood contact.	25-min PPT explanation + 10-min condom demonstration + 10-min Q&A.
Week 4	Comprehensive protection and healthy life	① HIV detection and consultation. ② Treatment and progress. ③ How to communicate health topics with family members. ④Available health resources in the village and surrounding areas. ⑤ Being a disseminator of health knowledge.	25-min PPT explanation + 10-min resource introduction + 10-min Q&A.

### 2.8 Measures

#### 2.8.1 Section 1: sociodemographic characteristics

Information on gender, age, education, marital status, and living arrangements was collected.

#### 2.8.2 Section 2: Comprehensive Capacity for HIV/AIDS Prevention

This study used the Comprehensive Capacity Assessment Scale for HIV/AIDS Prevention among Chinese Youth Students, developed by Gao et al. (30), as the foundational framework. Because the original scale was developed for Chinese youth students, it was culturally and age-appropriately adapted for rural older adults in this study. Therefore, the resulting instrument should be regarded as an adapted version with preliminary evidence of content validity and internal consistency in rural older adults, rather than a fully validated scale for this population.

The adaptation process involved four steps. First, through literature review and semi-structured interviews with eight rural older adults, items in the original scale that were difficult to understand or contextually inappropriate for older adults, such as references to students or ear piercing, were identified and modified or removed. Second, five experts from relevant fields, including two in nursing, two in infectious diseases, and one in geriatrics, evaluated the content validity of the revised instrument. The Scale-Level Content Validity Index was 0.92, and all Item-Level Content Validity Indices were ≥0.80, indicating good content relevance. Third, a pilot survey was conducted with 30 rural older adults who met the inclusion criteria. After item revision and pilot testing, the final adapted instrument comprised 27 items across four dimensions: knowledge, attitude, behavior, and skills. The original four-dimensional conceptual structure was retained after adaptation. The knowledge dimension included 8 items and used dichotomous scoring, with correct responses scored as 1 and incorrect or “unknown” responses scored as 0. The total score for the knowledge dimension ranged from 0 to 8. For the binary outcome of HIV knowledge awareness reported in Table 5, participants who answered at least 6 of the 8 knowledge items correctly, corresponding to a correct response rate of ≥75%, were classified as having HIV knowledge awareness. Participants who answered fewer than 6 items correctly were classified as not having HIV knowledge awareness. The knowledge awareness rate was calculated as the number of participants meeting the awareness criterion divided by the total number of participants in each group, multiplied by 100%. The attitude dimension included 7 items and used a 5-point Likert scale ranging from 1 = strongly disagree to 5 = strongly agree. The behavior dimension included 7 items and used a 5-point Likert scale ranging from 1 = strongly inconsistent with my behavior to 5 = strongly consistent with my behavior. The skills dimension included 5 items and used a 5-point Likert scale ranging from 1 = completely unable to 5 = completely able. The total score ranged from 19 to 103, with higher scores indicating stronger comprehensive capacity for HIV/AIDS prevention. Dimension scores were calculated by summing the items within each dimension. The overall Cronbach’s alpha coefficient was 0.85, with dimension-specific coefficients ranging from 0.78 to 0.82. HIV/AIDS prevention capacity was operationalized through the four dimensions shown in Table 6.

**Table 5:** Comparison of outcome indicators before and after intervention between the two groups (*n* = 97).

Items	Time point	Control group (*n* = 49)	Intervention group (*n* = 48)	Within-group comparison	Between-group comparison (post-intervention)
Knowledge [*n* (%)]	Pre	22 (44.8)	21 (43.7)	*χ*^2^ = 8.279, *p* = 0.004 (Control)	*χ*^2^ = 7.240, *p* = 0.012
	Post	36 (73.4)	45 (93.7)	*χ*^2^ = 27.927, *p* < 0.001 (Intervention)	Rate difference = 20.3% (6.2, 34.4%), Cramer’s *V* = 0.27
Attitude (Mean ± SD)	Pre	17.10 ± 3.38	16.33 ± 3.44	*t* = −3.027, *p* = 0.004 (Control)	*t* = 10.999, *p* < 0.001
	Post	20.18 ± 3.41	28.00 ± 3.59	*t* = −10.078, *p* < 0.001 (Intervention)	MD = 7.82 (95% CI: 6.45, 9.22), Cohen’s *d* = 2.23
Behavior (Mean ± SD)	Pre	14.84 ± 2.86	15.38 ± 3.35	*t* = −9.775, *p* < 0.001 (Control)	*t* = 3.541, *p* < 0.001
	Post	18.88 ± 4.49	22.31 ± 5.05	*t* = −11.377, *p* < 0.001 (Intervention)	MD = 3.43 (95% CI: 1.50, 5.36), Cohen’s *d* = 0.72
Skills (Mean ± SD)	Pre	11.18 ± 3.13	12.02 ± 3.23	*t* = −9.436, *p* < 0.001 (Control)	*t* = 4.140, *p* <0.001
	Post	13.45 ± 3.41	16.29 ± 3.35	*t* = −16.935, *p* < 0.001 (Intervention)	MD = 2.84 (95% CI: 1.48, 4.20), Cohen’s *d* = 0.84
Condom Use Self-Efficacy [Median (IQR)]	Pre	26.00 (22.00, 30.50)	28.00 (24.00, 34.00)	*Z* = −4.842, *p* < 0.001 (Control)	*Z* = −3.085, *p* = 0.002
	Post	31.00 (27.00, 35.00)	35.00 (31.25, 41.75)	*Z* = −5.825, *p* < 0.001 (Intervention)	Median difference = 4.00 (95% CI: 2.00, 7.00), Effect size *r* = 0.31

HIV knowledge awareness was defined as answering at least 6 of the 8 knowledge items correctly, corresponding to a correct response rate of ≥75%.

**Table 6:** Dimension definition.

Dimensions	Definitions	Number of items	Scoring	Example item
Knowledge	Accurate understanding of HIV transmission routes, prevention methods, and common misconceptions	8	Dichotomous scoring (correct = 1, incorrect/unknown = 0)	Can HIV be transmitted through mosquito bites? (Correct answer: No)
Attitude	Beliefs and social attitudes toward HIV, including stigma-related perceptions and risk perception	7	Likert 5-point scale (1 = strongly disagree, 5 = strongly agree)	If a friend or neighbor is infected with HIV, I am willing to maintain normal interactions with them
Behavior	Implementation of HIV preventive behaviors, including condom use and testing-seeking behavior	7	Likert 5-point scale (1 = strongly does not conform, 5 = strongly conforms)	If I suspect I might be infected with HIV, I will immediately go to a township health center or CDC for consultation and testing
Skill	Capacity to perform HIV preventive behaviors, including condom use skills and negotiation skills for safe behaviors	5	Likert 5-point scale (1 = strongly unable, 5 = strongly able)	I can accurately identify high-risk behaviors that are prone to HIV infection (e.g., sharing needles for drug use, having multiple sexual partners)

#### 2.8.3 Section 3: Condom Self-Efficacy Scale

The Chinese version of the Condom Self-Efficacy Scale (CSES), developed by Hanna (31) and translated and culturally adapted by Zhao et al. (32), was employed in this study. The scale comprises 14 items across three dimensions: persistent use, correct use, and negotiation of use. A Likert 5-point rating system was adopted (1 = very uncertain, 5 = very certain), with total scores ranging from 14 to 70. Higher scores indicate better condom use self-efficacy.

This Chinese version has demonstrated good reliability and validity in studies targeting rural older adult populations and adolescents in China (33, 34), with a Cronbach’s ɑ coefficient of 0.96. In the present study, the Cronbach’s ɑ coefficient for the scale was 0.93.

### 2.9 Statistical analysis

Data were analyzed using SPSS version 21.0. Categorical variables were expressed as percentages and compared using the chi-square test. Continuous variables were presented as mean ± standard deviation (SD) and compared using independent-samples t-tests. A two-sided *p*-value < 0.05 was considered statistically significant. Data analysts were blinded to group allocation. Although no statistically significant baseline differences were observed between the two groups, ANCOVA was conducted as a supplementary sensitivity analysis to adjust for baseline outcome scores and improve the precision of the estimated intervention effects. Post-intervention scores were entered as dependent variables, group assignment was entered as the independent variable, and the corresponding baseline scores were entered as covariates. No subgroup analyses by sex or age were conducted because the sample size was limited and the study was not powered to detect subgroup-specific intervention effects.

## 3 Results

### 3.1 Description of study samples

During the intervention period, one participant in the control group withdrew for personal reasons, and two participants in the intervention group were lost to follow-up due to relocation or employment changes. A total of 97 participants completed the study (intervention group: *n* = 48; control group: *n* = 49) (Figure 3). No statistically significant differences were observed between the two groups in baseline sociodemographic characteristics or outcome measures prior to the intervention (*p* > 0.05) (Tables 1, 7), indicating good baseline comparability.

**Figure 3:**
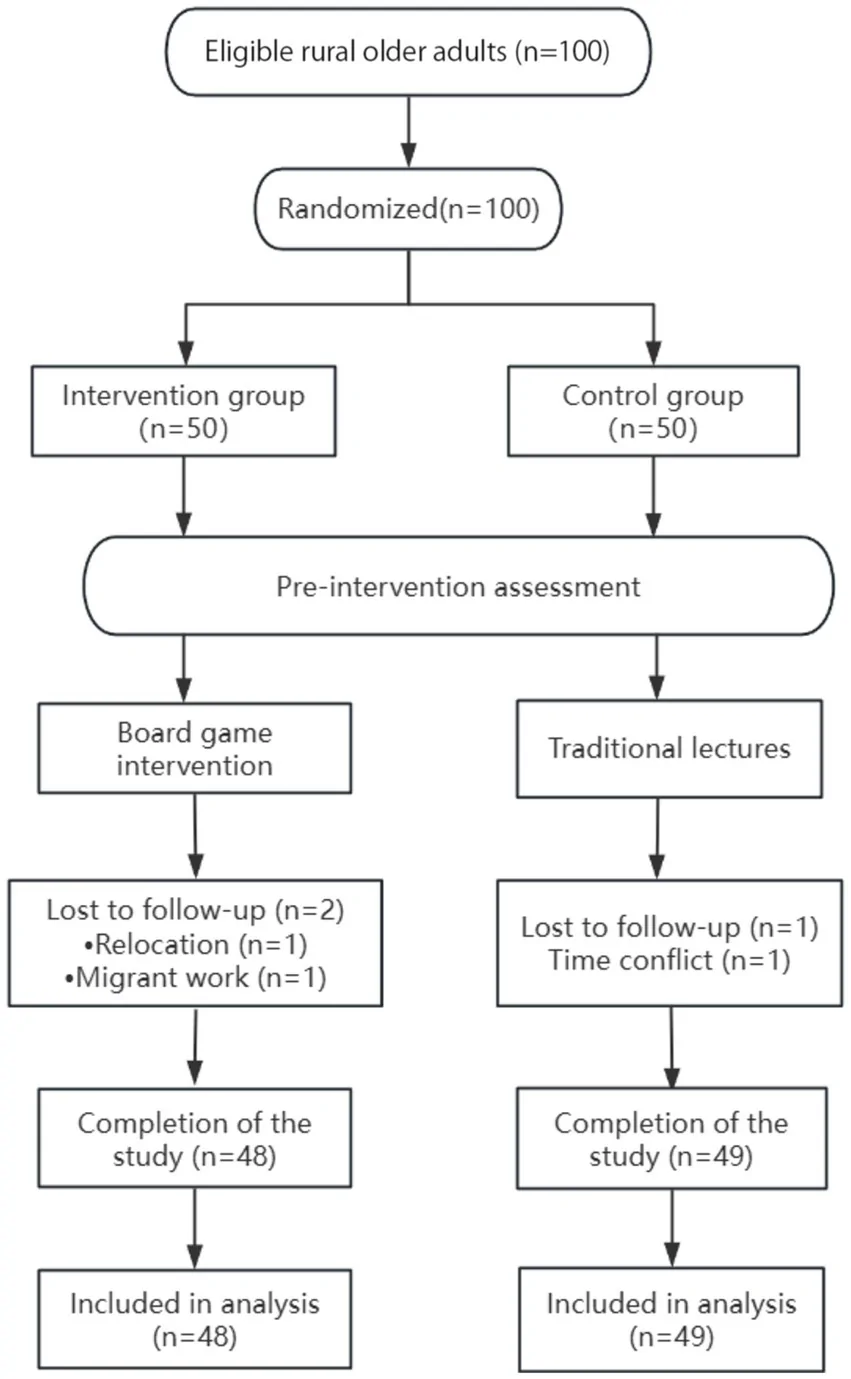
CONSORT diagram.

**Table 7:** Comparison of indicators between the two groups before the intervention (*n* = 97).

Items	Intervention group (*n* = 48)	Control group (*n* = 49)	Mean difference (95% CI)	*Cohen’s d*	*t/χ^p^/z*	*p*
Knowledge [*n* (%)]	21 (43.7)	22 (44.8)	−5.0% (−22.1, 12.1%)	0.10	0.013	0.909
Attitude (Mean ± SD)	16.33 ± 3.44	17.10 ± 3.38	0.02 (−0.32, 0.36)	0.02	−1.111	0.269
Behavior (Mean ± SD)	15.38 ± 3.35	14.84 ± 2.86	−0.02 (−0.31, 0.27)	0.03	0.852	0.396
Skills (Mean ± SD)	12.02 ± 3.23	11.18 ± 3.13	0.11 (−0.09, 0.31)	0.23	1.297	0.198
Self-efficacy for condom use [Median (IQR)]	28.00 (24.00, 34.00)	26.00 (22.00, 30.50)	0.10 (−0.13, 0.33)	0.18	−1.284	0.199

### 3.2 Comparison of indicators between the two groups before and after the intervention

Following the intervention, scores across all dimensions—HIV-related knowledge, attitudes, behaviors, and skills—significantly increased in both groups compared with baseline (*p* < 0.05).

Between-group comparisons demonstrated that the intervention group achieved significantly higher scores across all dimensions than the control group (*p* < 0.05) (Table 5).

### 3.3 ANCOVA results

Although no statistically significant baseline differences were observed between the two groups, ANCOVA was conducted as a supplementary sensitivity analysis to adjust for baseline outcome scores and improve the precision of the estimated intervention effects. For each outcome indicator, the post-intervention score was entered as the dependent variable, group assignment was entered as the independent variable, and the corresponding baseline score was entered as the covariate. This analysis was not intended to replace the primary analysis based on independent-samples t-tests, but rather to examine the robustness of the intervention effects after adjustment for baseline outcome scores. The ANCOVA results were consistent with those of the primary analysis,supporting the robustness of the study findings (Table 8).

**Table 8:** ANCOVA results for outcome indicators adjusted for baseline scores.

Outcome	Adjusted mean (95% CI)	Adjusted mean difference (95% CI)	*F*	*p*	Partial *η*^2^
Attitude			109.313	<0.001	0.538
Intervention group	27.93 (26.91, 28.95)				
Control group	20.25 (19.25, 21.26)	7.67 (6.22, 9.13)			
Behavior			15.276	<0.001	0.140
Intervention group	22.04 (21.00, 23.08)				
Control group	19.15 (18.11, 20.18)	2.90 (1.42, 4.37)			
Skills			35.244	<0.001	0.273
Intervention group	15.90 (15.41, 16.39)				
Control group	13.83 (13.35, 14.32)	2.07 (1.38, 2.77)			
Condom self-efficacy			14.383	<0.001	0.133
Intervention group	36.42 (34.82, 38.01)				
Control group	32.12 (30.54, 33.70)	4.30 (2.05, 6.55)			

## 4 Discussion

This study evaluated the effectiveness of a board game-based health education intervention in enhancing HIV/AIDS-related knowledge, attitudes, behavioral skills, and condom use self-efficacy among rural older adults in Hunan Province. Three key findings emerged. First, the board game intervention substantially improved HIV/AIDS knowledge awareness among rural older adults. Second, participants in the intervention group demonstrated greater improvements in HIV/AIDS-related attitudes, preventive behaviors, and behavioral skills than those receiving lecture-based education. Third, the intervention also enhanced condom use self-efficacy, suggesting that board game-based health education may help strengthen both cognitive and behavioral readiness for HIV prevention. Collectively, these findings suggest that board game-based health education is a feasible and potentially effective approach for HIV/AIDS prevention among rural older adult populations.

### 4.1 Mechanisms of intervention effectiveness

Health education remains a fundamental strategy for reducing high-risk behaviors and promoting preventive measures. In recent years, board game-based learning has emerged as an innovative approach to improving engagement and learning outcomes in health education. By integrating structured gameplay with educational content, board game-based interventions create an interactive learning environment that supports knowledge acquisition, risk awareness, and behavioral skill development (22, 23). Compared with traditional lecture-style education, the board game intervention in this study may have exerted its effects through the following mechanisms.

First, it reduced cognitive barriers and enhanced learning motivation. Rural older adults individuals often exhibit low engagement with didactic, one-way lectures and face significant obstacles in accessing digital health information due to limited digital literacy (35, 36). As a familiar and culturally acceptable form of entertainment, board games may help reduce psychological resistance and cognitive burden. From the perspective of game-based learning frameworks, clear rules, goals, progression, competition, cooperation, and immediate feedback can transform passive information reception into active learning tasks. In this study, game elements such as dice rolling, movement on the board, card-based questions, team competition, and immediate feedback encouraged participants to engage repeatedly with HIV-related information. This process made abstract health knowledge more concrete and easier to understand, thereby improving comprehension and knowledge retention.

Second, contextualized design facilitated knowledge-to-behavior translation. The board game incorporated real-life scenarios closely relevant to rural older adults, such as identifying high-risk behaviors or navigating informal medical practices. Through repeated exposure to these scenarios in a simulated environment, participants were able to transform abstract knowledge into practical risk awareness and coping strategies. This experiential learning process strengthened the connection between knowledge and action (37).

This mechanism aligns closely with the core constructs of the Health Belief Model. This model posits that an individual‘s likelihood of adopting a health behavior depends on their perception of disease threat (perceived susceptibility and perceived severity) as well as their assessment of the benefits and barriers to taking action (38). In this study, risk-related and scenario-based content was embedded within Attitude Cards and Behavior Cards rather than presented as a separate “risk card” category. Attitude Cards guided participants to reflect on HIV-related risk perception, stigma, and prevention-related attitudes, whereas Behavior Cards focused on feasible preventive actions such as condom use and HIV testing. For example, card content related to “sharing razors” and “informal medical practices” helped participants recognize everyday HIV-related risks and strengthened perceived susceptibility. Meanwhile, Behavior Cards presenting preventive actions helped participants understand the benefits and feasibility of prevention, thereby reducing perceived barriers to preventive behaviors (39). Through these pathways, the board game intervention effectively facilitated the reconstruction of participants’ health beliefs, thereby promoting the behavioral translation from “knowing” to “doing.”

Third, peer interaction within the gaming environment promoted attitudinal change. Peer discussion was operationalized through within-team deliberation before responding to card questions, facilitator-guided discussion after feedback on Attitude Cards and Behavior Cards, and the “Your turn to share” activity during the summary and reinforcement phase. These structured opportunities for discussion, debate, and reflection enabled participants to confront HIV-related misconceptions and social stigma in a relatively relaxed group environment. Compared with traditional top-down education, peer-based interaction provided a more persuasive and resonant learning environment. Additionally, competitive and cooperative elements enhanced participants’ enjoyment and engagement, contributing to improved adherence to the intervention.

This finding can be explained by the mechanism of “observational learning” within Social Cognitive Theory. This theory posits that individuals learn not only through their own direct experiences but also by observing the behaviors of others and the consequences that follow, thereby acquiring new cognitions, attitudes, and behavioral patterns. In the group game process of this study, when a participant expressed a non-stigmatizing attitude during discussion of an Attitude Card, such as “People living with HIV should not be isolated; daily contact does not transmit HIV,” and received peer approval and facilitator affirmation, other participants could reassess and adjust their own prejudiced attitudes by observing this positive interaction and its outcomes. This attitude formation process, grounded in authentic social interaction, is more likely to evoke cognitive resonance and emotional identification than one-way education that relies solely on expert lectures. Moreover, because peers share similar age backgrounds, life experiences, and sociocultural environments, their behaviors and opinions often carry greater credibility and imitability than authoritative instruction (18). Peers’ correct answers, positive attitudes, and cooperative behaviors provide concrete cognitive references and behavioral models for other participants, helping to reduce HIV-related stigma and enhance perceived social support. Previous studies have shown that HIV interventions based on peer interaction and social support can effectively reduce stigma, improve psychological adjustment, and promote health behavior change (40). Furthermore, research on older adults living with HIV has indicated that social support and positive social interaction are important protective factors for alleviating stigma and enhancing health behaviors (41). These findings further support the important role of the peer interaction mechanism in facilitating attitude improvement observed in this study.

### 4.2 Comparison with previous studies

The magnitude of improvement in HIV-related knowledge observed in this study aligns with findings from prior research on board game interventions in other health domains. Gauthier et al. (42) conducted a systematic review and meta-analysis of 21 studies, demonstrating the general effectiveness of board game interventions in facilitating knowledge acquisition. This study extends this evidence to HIV prevention among older adults, a population that has been underrepresented in previous game-based learning research.

The greater attitudinal improvements in the intervention group can be understood through the combined lens of game-based learning and Social Cognitive Theory. By transforming passive learning into active exploration, the board game format effectively addressed the emotional and social dimensions of health behaviors. The relaxed, interactive setting enabled participants to openly discuss sensitive topics such as sexual health and HIV-related stigma, which are often difficult to address in traditional educational settings (43). Structured peer discussion during Attitude Cards, Behavior Cards, and the “Your turn to share” activity further facilitated reflection and attitudinal shifts, contributing to reduced stigma (44). This finding is consistent with Tang et al. (45), who studied game-based HIV prevention interventions for adolescents, suggesting that game-based strategies can effectively improve attitudes across age groups.

In terms of behavioral intentions and skills, the board game created a safe and supportive environment for repeated practice of preventive behaviors, such as refusing high-risk situations and correctly using condoms. This aligns with social cognitive theory, which emphasizes the role of observational learning and mastery experiences in building self-efficacy (39). The structured exercises embedded in the game enhanced skill acquisition and confidence. Notably, the intervention effectively addressed the widespread “knowledge-attitude-practice” gap among rural older adults. Traditional education often leads to awareness improvements without translating into behavioral competence. In contrast, Behavior Cards with scenario-based content required active, step-by-step practice, facilitating knowledge-to-skill transformation. This experiential learning approach has been shown to significantly improve skill mastery compared to knowledge-based instruction alone (46, 47).

### 4.3 Study limitations

This study has several limitations. First, the intervention lasted for a relatively short period (4 weeks) and lacked long-term follow-up, limiting our ability to assess the sustainability of the intervention effects. It remains unclear whether the observed improvements in knowledge, attitudes, and self-efficacy immediately after the intervention would persist or translate into actual behavioral changes in real-world settings. Second, the sample was drawn from two villages in Hunan Province, which may restrict the generalizability of the findings to other rural areas in China or to countries with different cultural and socioeconomic contexts. Third, subgroup analyses by sex or age were not conducted because of the limited sample size and insufficient statistical power for subgroup-specific comparisons. Future studies with larger and more diverse samples should examine whether intervention effects differ across demographic subgroups. Fourth, the Comprehensive Capacity Assessment Scale for HIV/AIDS Prevention used in this study was adapted from an instrument originally developed for Chinese youth students. Although item revision, expert content validity assessment, pilot testing, and internal consistency analysis were conducted, no exploratory or confirmatory factor analysis was performed. Therefore, the structural validity of the adapted instrument among rural older adults remains to be further examined in future studies with larger samples. Finally, given the nature of the intervention, blinding of participants and facilitators was not feasible, which may have introduced performance bias. However, the data analysts were blinded to group allocation, which helped mitigate detection bias.

### 4.4 Implications for practice and future research

Despite the aforementioned limitations, the findings of this study hold significant implications for HIV prevention practice and future research. From a practical perspective, the board game intervention offers a low-cost, low-technology, and culturally adaptable approach that can be integrated into existing community health education programs in rural areas. The intervention can be implemented by trained rural health workers, ensuring sustainability within China’s existing primary healthcare infrastructure. Future implementation studies should examine the feasibility, acceptability, and cost-effectiveness of scaling up this intervention to other rural regions.

From a research standpoint, future studies could explore several directions. First, longitudinal research with longer follow-up periods (e.g., 6–12 months) is needed to assess the durability of intervention effects and their translation into real-world behavioral outcomes, such as consistent condom use and HIV testing uptake. Second, randomized controlled trials with larger, more diverse samples would enhance generalizability. Third, future studies should further evaluate the psychometric properties of the adapted Comprehensive Capacity Assessment Scale for HIV/AIDS Prevention among rural older adults, including its structural validity through exploratory or confirmatory factor analysis. Fourth, board games could be adapted for other health topics (e.g., other infectious diseases, chronic disease self-management) and other populations (e.g., adolescents, migrant workers) could maximize their public health impact.

## 5 Conclusion

This study suggests that a board game-based health education intervention can effectively enhance HIV-related knowledge, attitudes, behavioral skills, and condom use self-efficacy among rural older adults. Compared with lecture-based education, the board game-based intervention produced greater improvements across the measured outcomes, including HIV knowledge awareness, HIV-related attitudes, behavioral skills, and condom use self-efficacy. The pilot study and cognitive adaptations tailored to rural older adults provided preliminary support for the feasibility and acceptability of this intervention in the target population.

Despite the limitations discussed above, the board game intervention represents a promising, low-cost, and scalable strategy for HIV prevention among rural older adults. It can serve as a valuable addition to existing community health education programs, especially in resource-limited settings where digital or technology-based interventions are not feasible. As the global population ages and the HIV burden among older adults continues to rise, innovative, age-appropriate, and culturally tailored health education approaches are increasingly needed to meet the health needs of this growing population.

## Data Availability

The data supporting the findings of this study are available from the corresponding author upon reasonable request.
