# Supplementary material for: Board game-based education for HIV prevention among rural older adults: a quasi-experimental study
Source: Front Public Health. 2026 Oct 1;14:1834731. doi: 10.3389/fpubh.2026.1834731 (PMC13669154; doi:10.3389/fpubh.2026.1834731)
Supplement: Supplementary file 1 [file Data_Sheet_1.pdf]

HIV/AIDS Prevention Education Card Set for Rural Elderly Population

This supplementary file contains three sets of educational cards designed to be used alongside the board game intervention:

Knowledge Cards: These cards feature true/false or multiple-choice questions covering HIV transmission routes, prevention methods, and common misconceptions. They are designed to improve participants' factual understanding of HIV/AIDS in an accessible format suitable for older adults with varying literacy levels.

Attitude Cards : These cards present short, realistic scenarios or statements about stigma, discrimination, and misconceptions regarding people living with HIV. They are intended to facilitate group discussions and promote positive, empathetic attitudes towards affected populations.

Behavioral Cards: These cards describe risk scenarios and protective actions in daily life. Participants are asked to identify risks or choose appropriate protective behaviors, reinforcing practical skills for HIV prevention.

Purpose: These cards serve as the core interactive components of the board game intervention, translating abstract health knowledge into tangible, discussion-based learning experiences for rural elderly participants. The content has been culturally adapted to ensure relevance and comprehension.

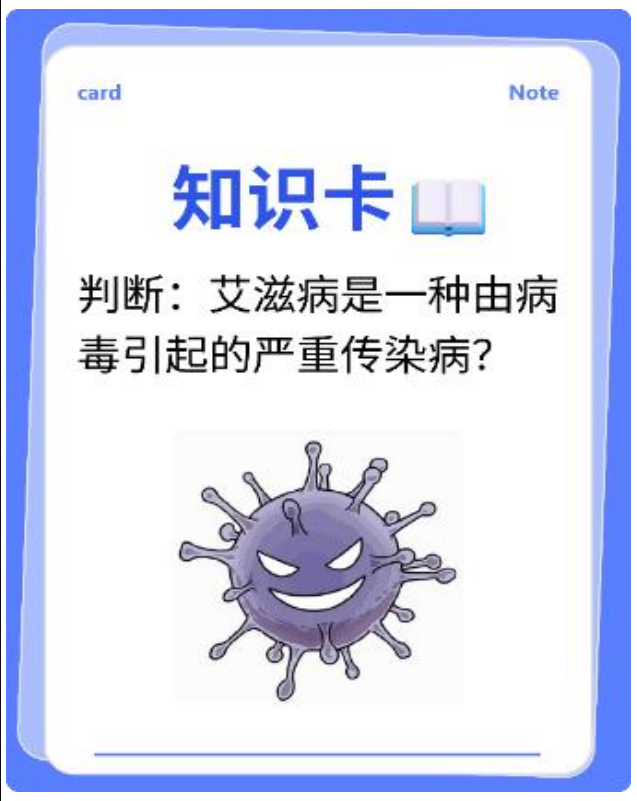	Knowledge Card<br>True or False: AIDS is a serious infectious disease caused by a virus?	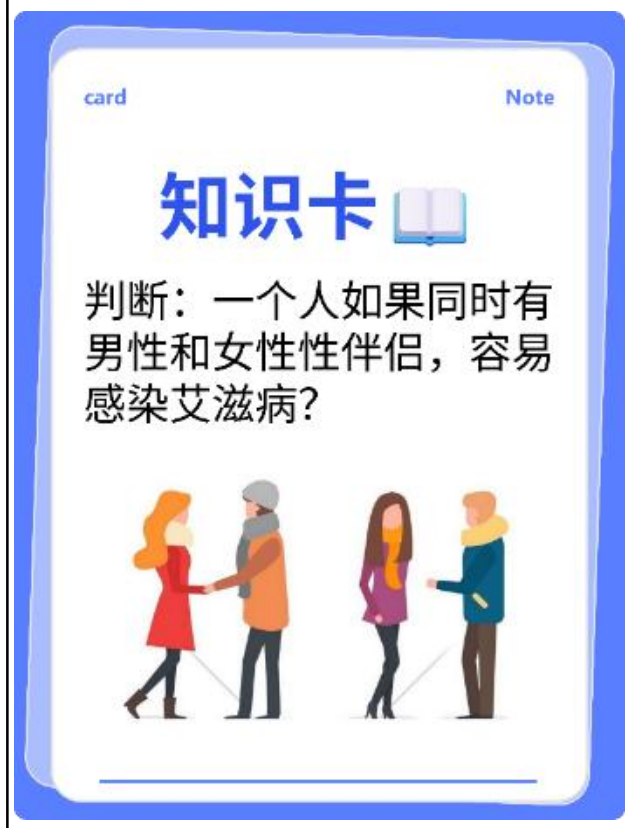	Knowledge Card<br>True or False: Is a person more likely to contract HIV if they have both male and female sexual partners?
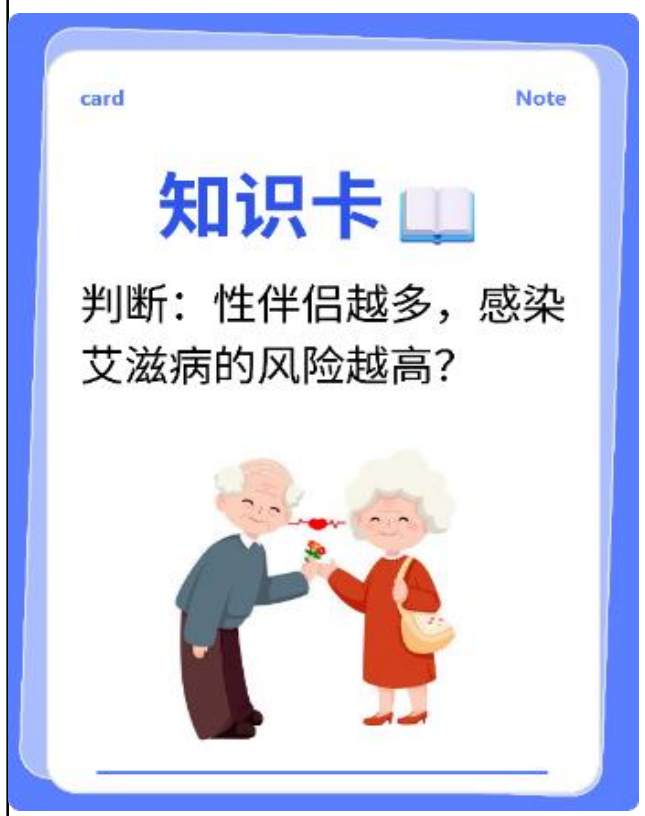	Knowledge Card<br>True or False: The more sexual partners you have, the higher your risk of contracting HIV?	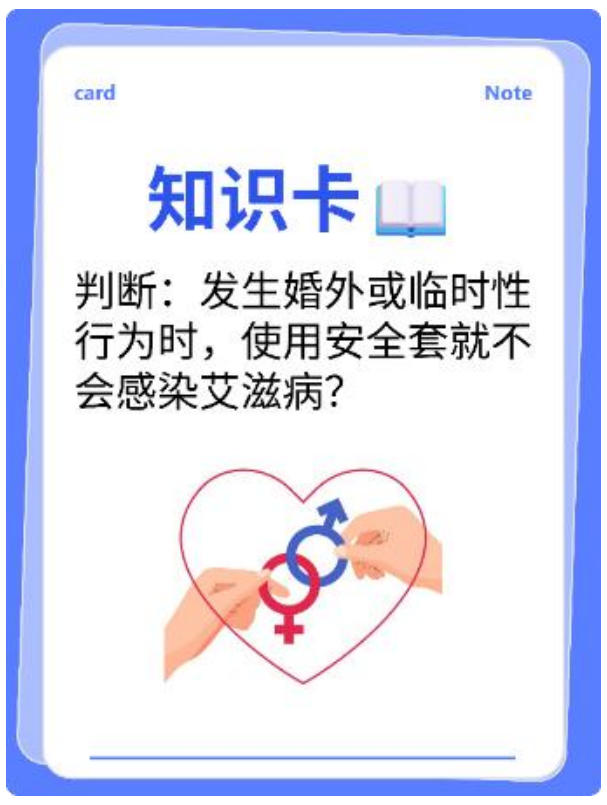	Knowledge Card<br>True or False: Using a condom during extramarital or casual sex prevents HIV infection?
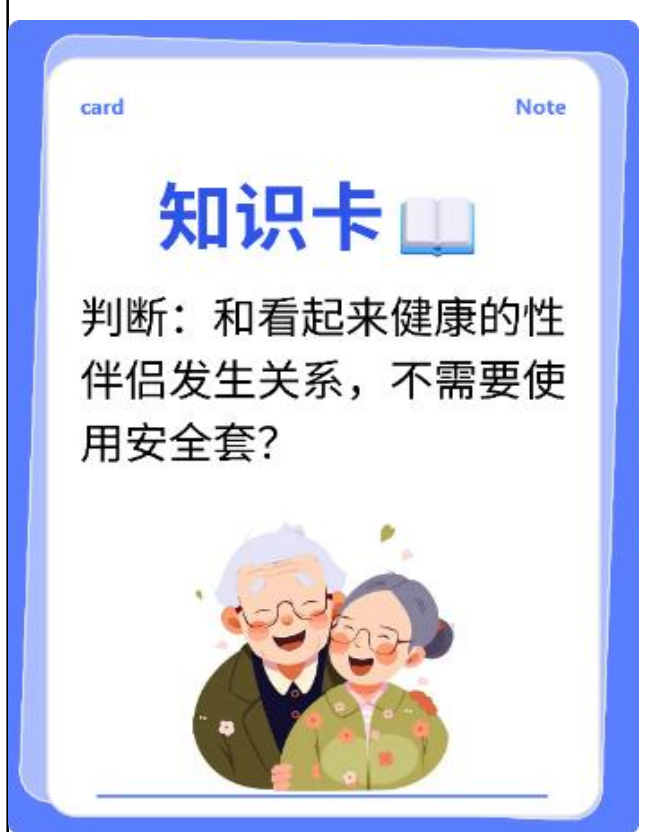	Knowledge Card<br>True or False: You don't need to use condoms when having sex with a partner who looks healthy?	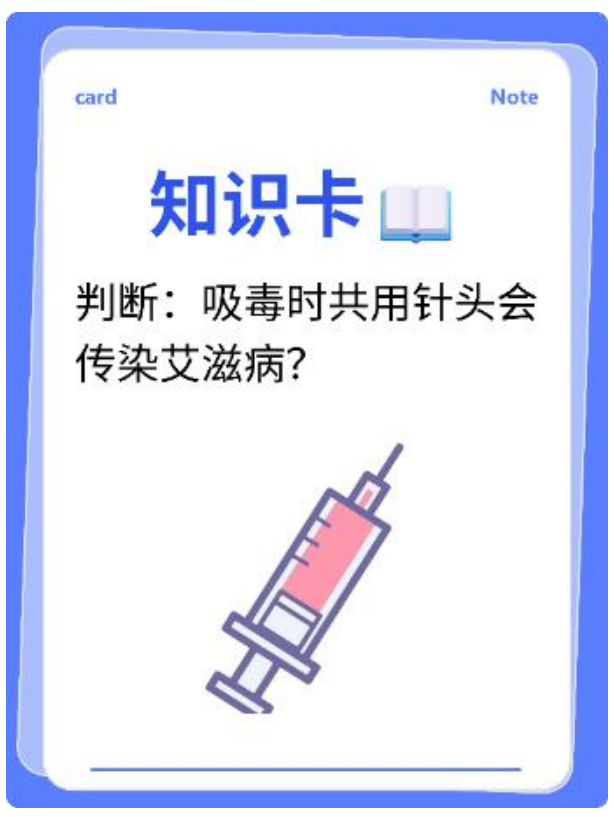	Knowledge Card<br>True or False: Sharing needles when injecting drugs can transmit HIV?
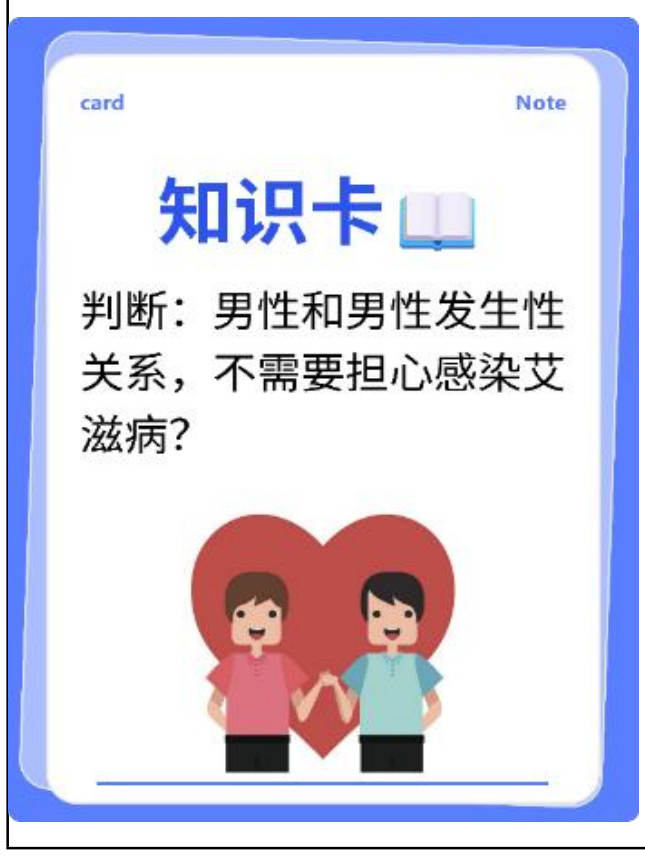	Knowledge Card<br>True or False: Men who have sex with men do not need to worry about contracting HIV?	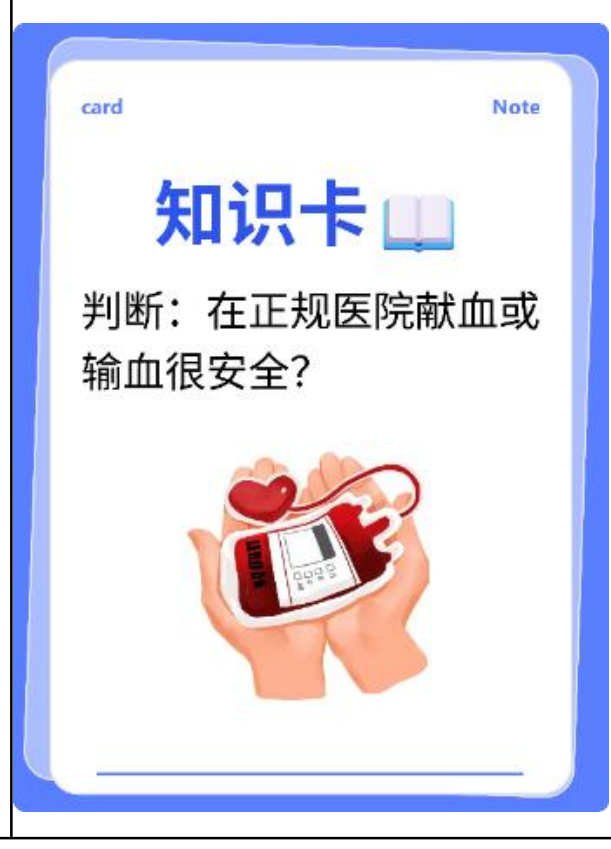	Knowledge Card<br>True or False: Is it safe to donate or receive blood at a formal hospital?

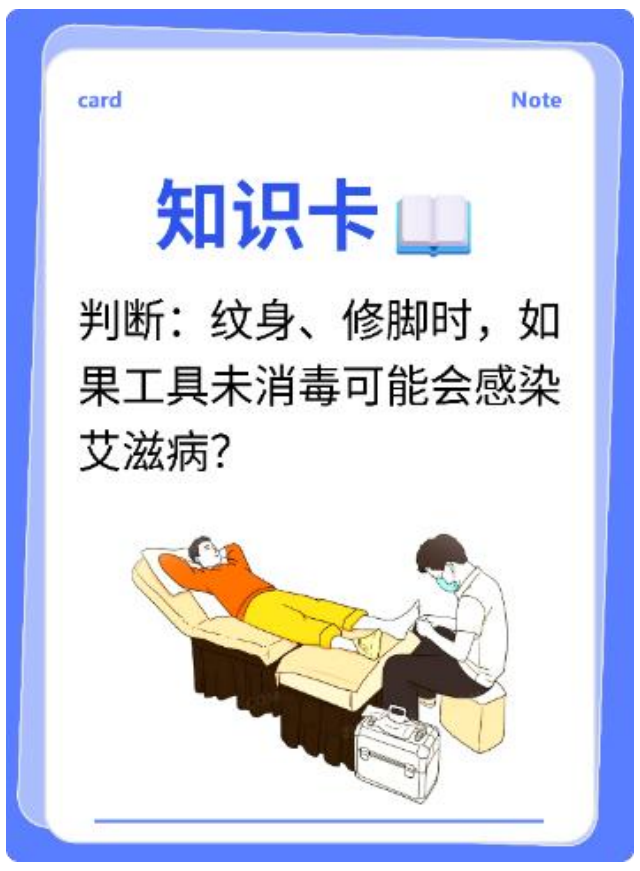	Knowledge Card<br>True or False: Can HIV infection occur if tools are not sterilized during tattooing or pedicures?	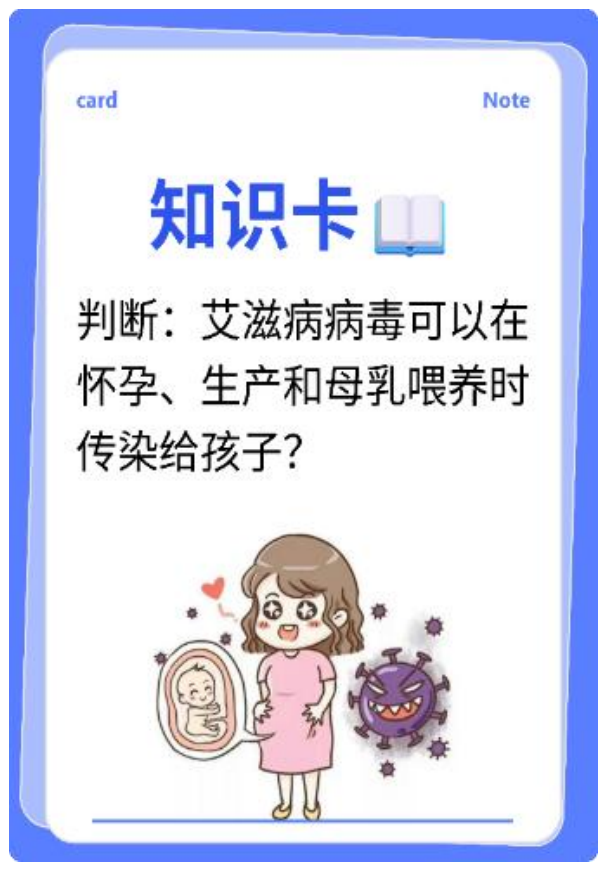	Knowledge Card<br>True or False: Can HIV be transmitted to a child during pregnancy, childbirth, or breastfeeding?
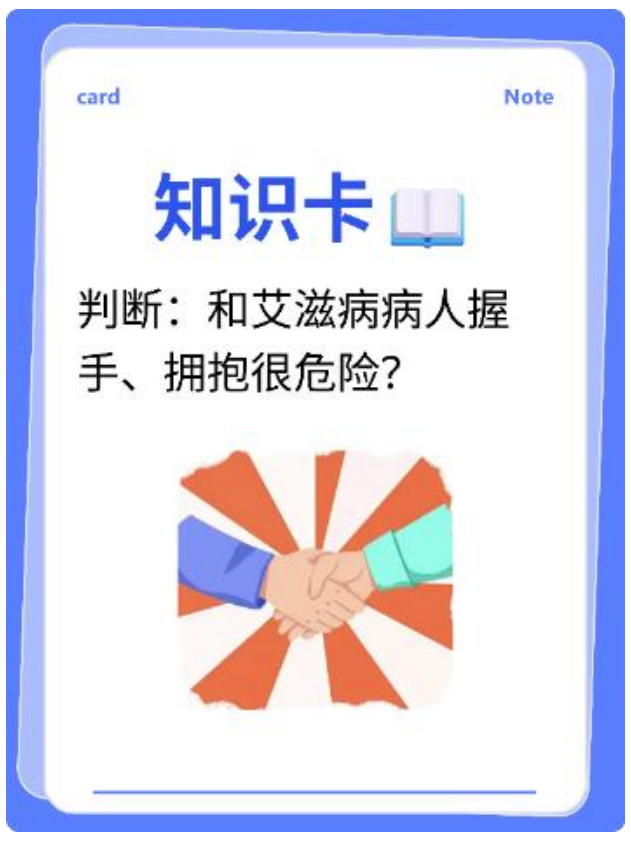	Knowledge Card<br>True or False: Is it dangerous to shake hands or hug someone with AIDS?	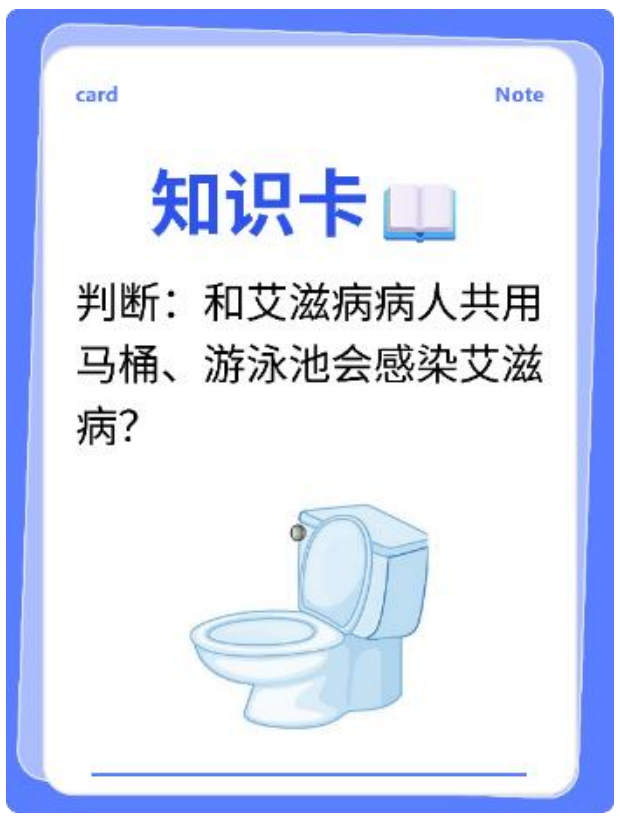	Knowledge Card<br>True or False: Can you contract HIV by sharing a toilet or swimming pool with someone who has AIDS?
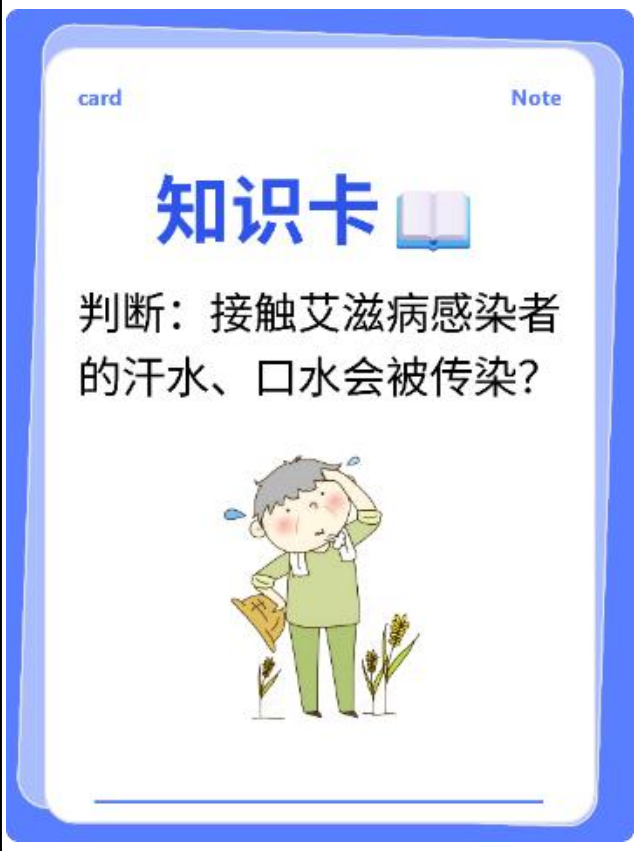	Knowledge Card<br>True or False: Can you get infected by coming into contact with the sweat or saliva of someone with HIV?	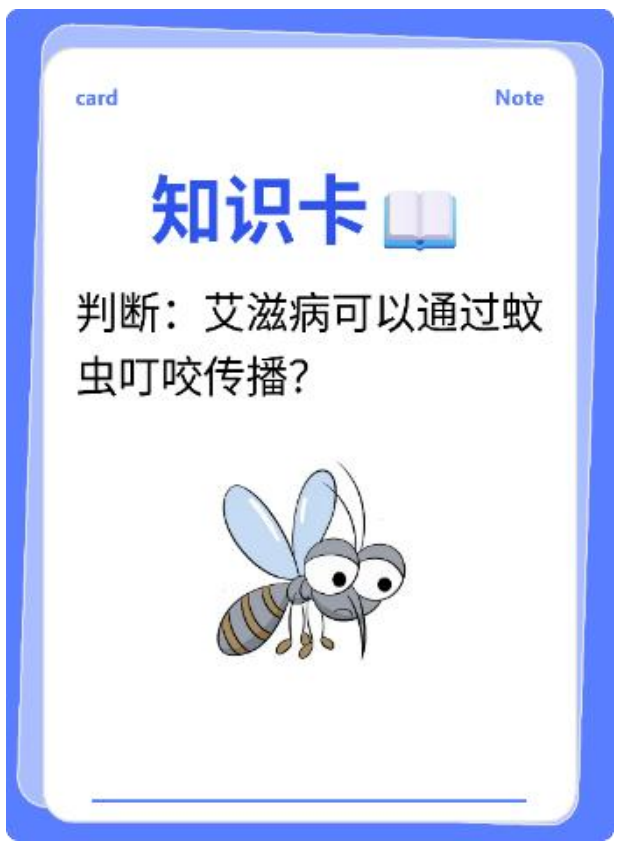	Knowledge Card<br>True or False: Can AIDS be transmitted through mosquito bites?
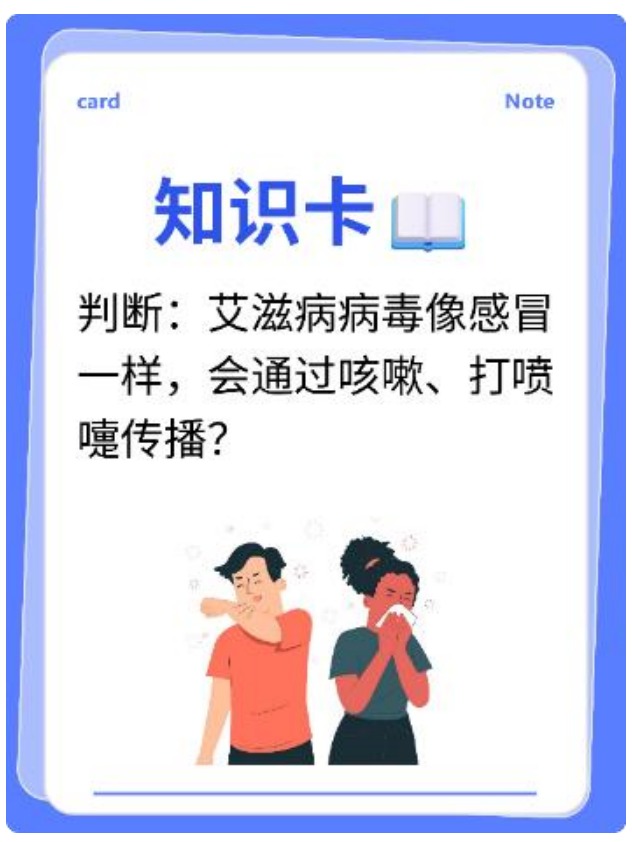	Knowledge Card<br>True or False: Does HIV spread through coughing and sneezing like the common cold?	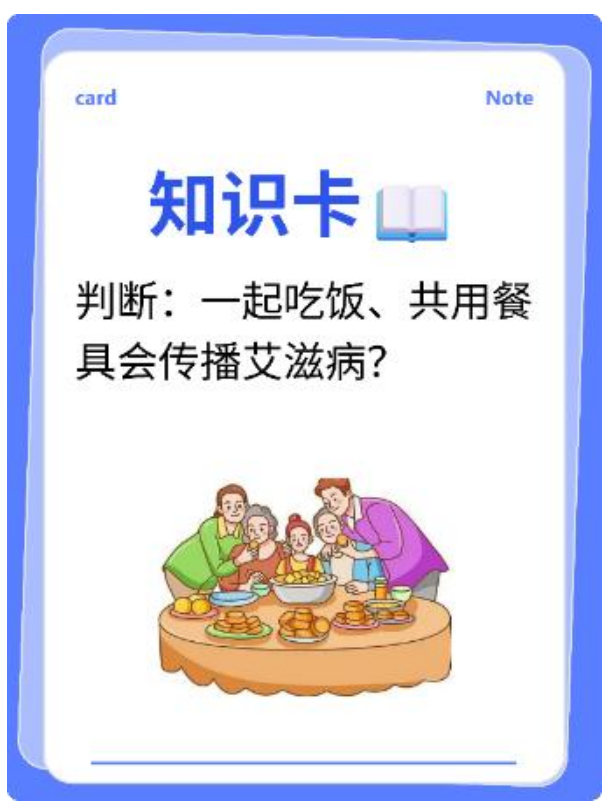	Knowledge Card<br>True or False: Can HIV be transmitted by eating together or sharing utensils?

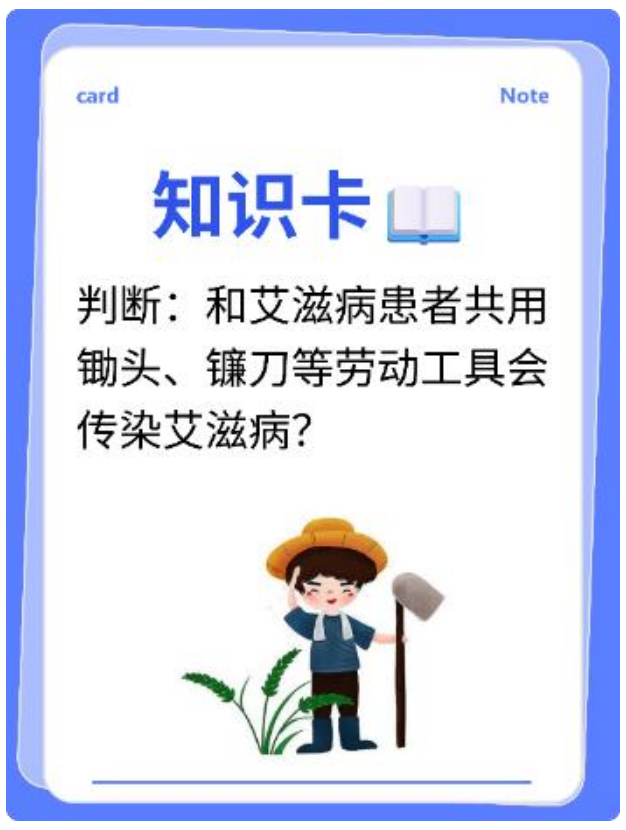	Knowledge Card<br>True or False: Can HIV be transmitted by sharing work tools with an HIV-positive person?	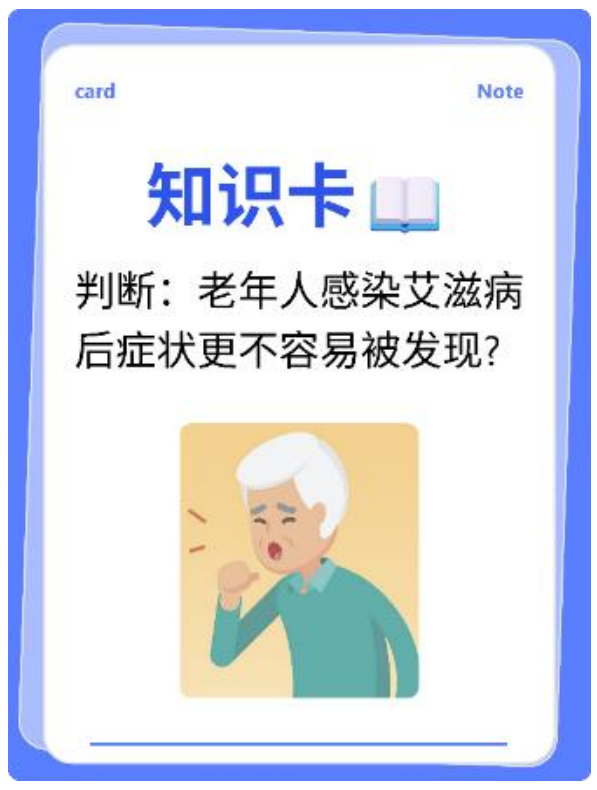	Knowledge Card<br>True or False: Are symptoms less likely to be detected in older adults after HIV infection?
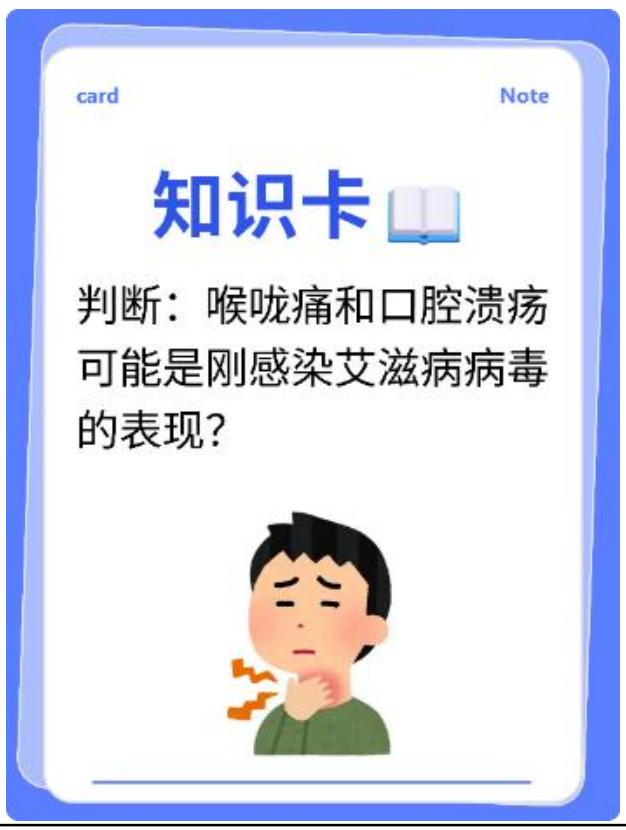	Knowledge Card<br>True or False: Can a sore throat and mouth ulcers be early symptoms of HIV infection?	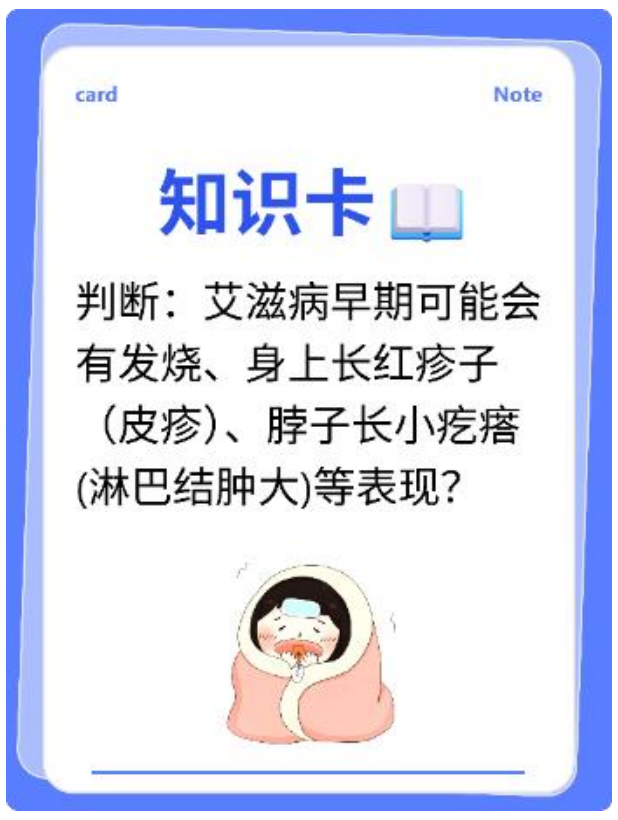	Knowledge Card<br>True or False: Early-stage AIDS may present with symptoms such as fever and rash?
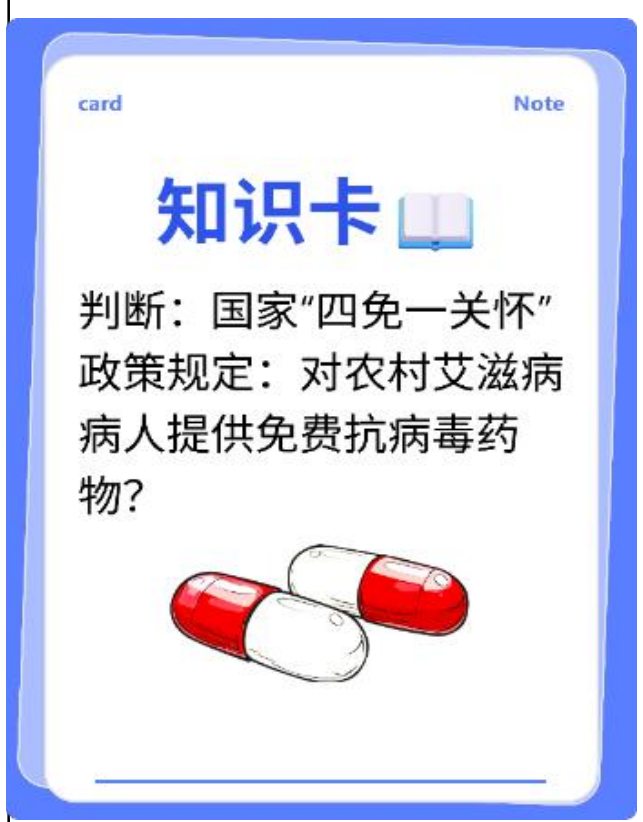	Knowledge Card<br>True or False: National policy stipulates that free antiviral drugs are provided to rural AIDS patients?	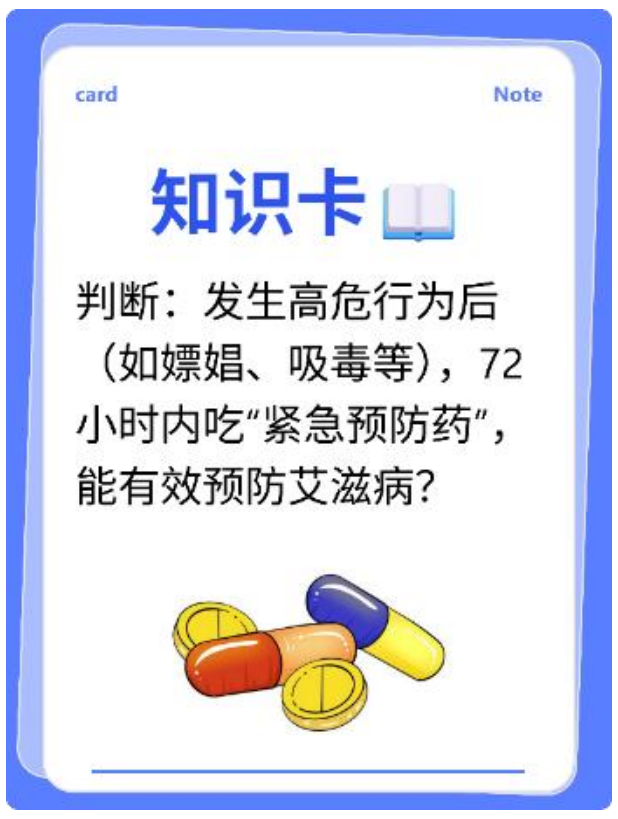	Knowledge Card<br>True or False: Taking "emergency prevention medication" within 72 hours after engaging in high-risk behaviors (such as prostitution or drug use) can effectively prevent HIV infection?
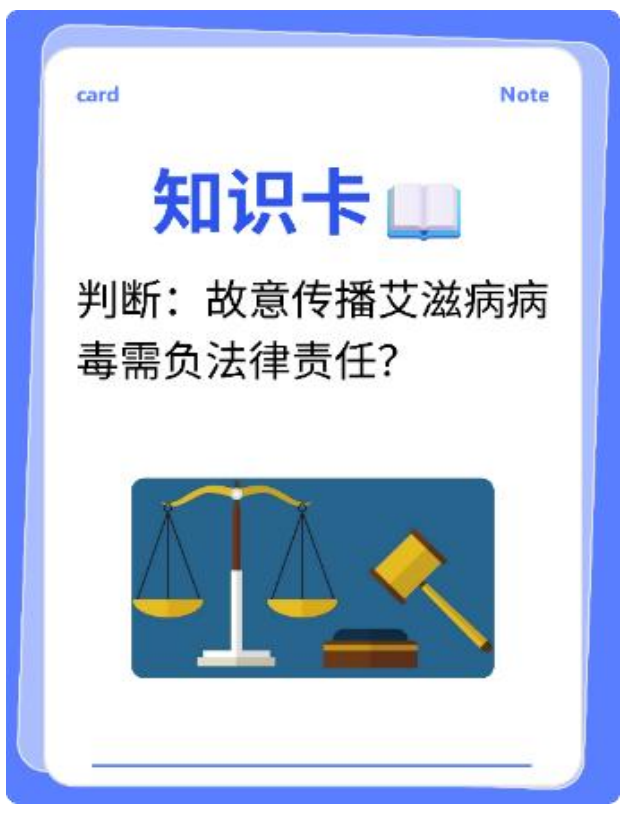	Knowledge Card<br>True or False: Is there legal liability for intentionally transmitting HIV?	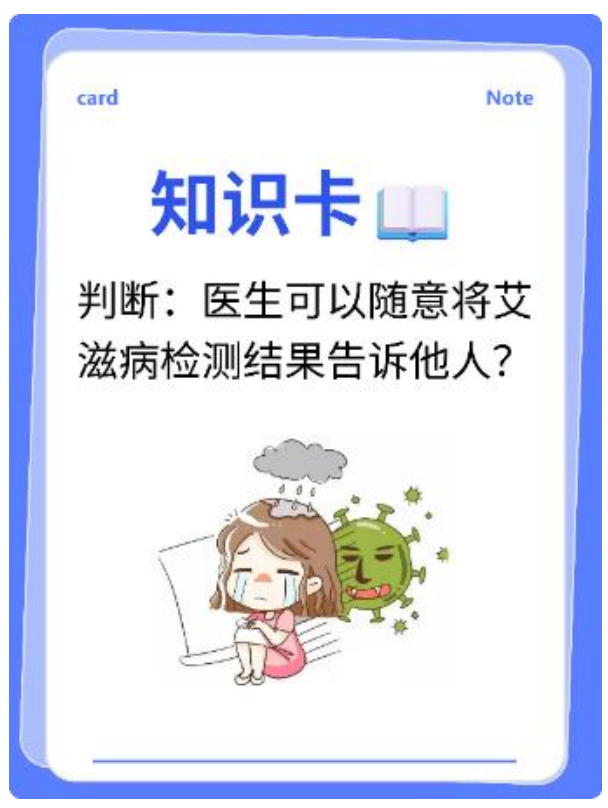	Knowledge Card<br>True or False: Can doctors freely disclose HIV test results to others?

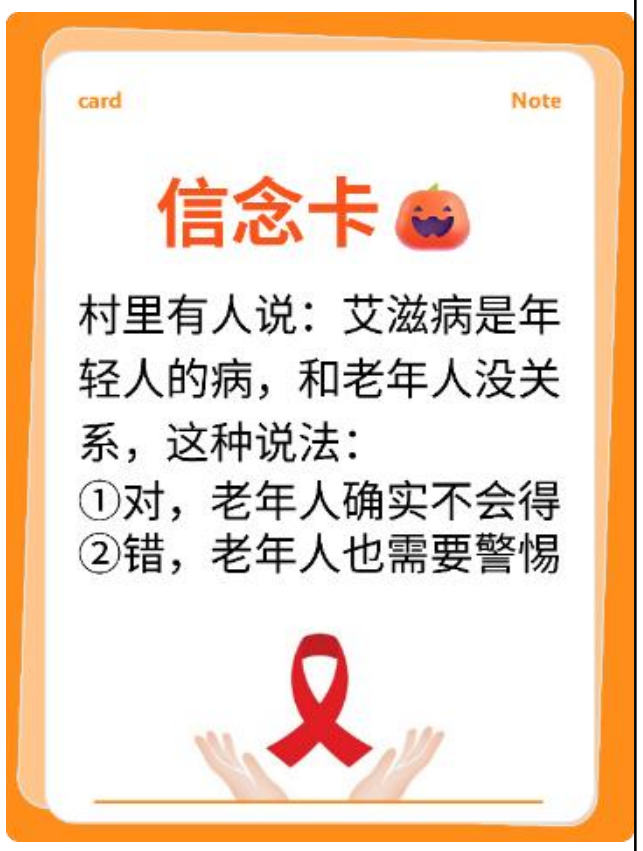	Attitude Card<br>Some villagers say: AIDS is a disease of the young and has nothing to do with the elderly. Is this statement?<br>① True. The elderly cannot be infected.<br>② False. The elderly need to be vigilant.	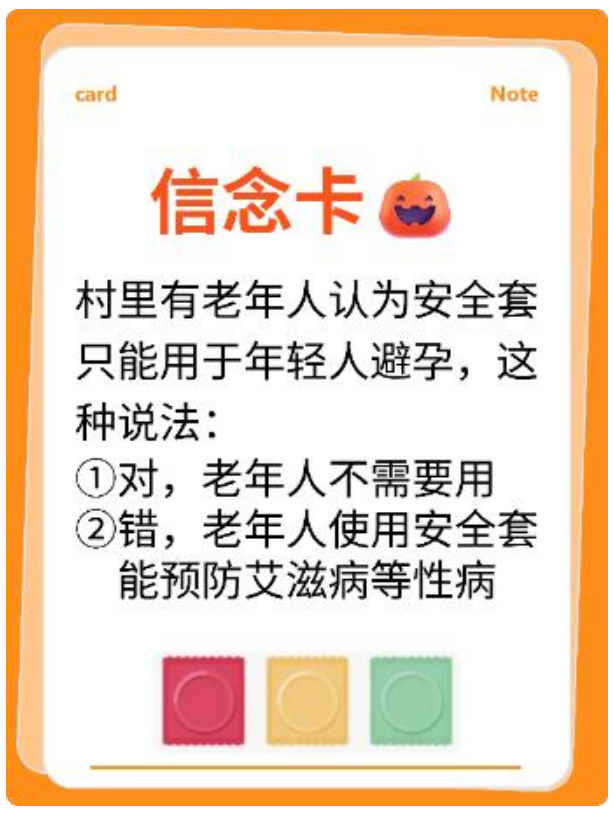	Attitude Card<br>Some older villagers believe condoms are only for young people's contraception. Is this belief:<br>① Correct – older adults don't need condoms<br>② Incorrect – condom use prevents HIV/STIs among older adults too
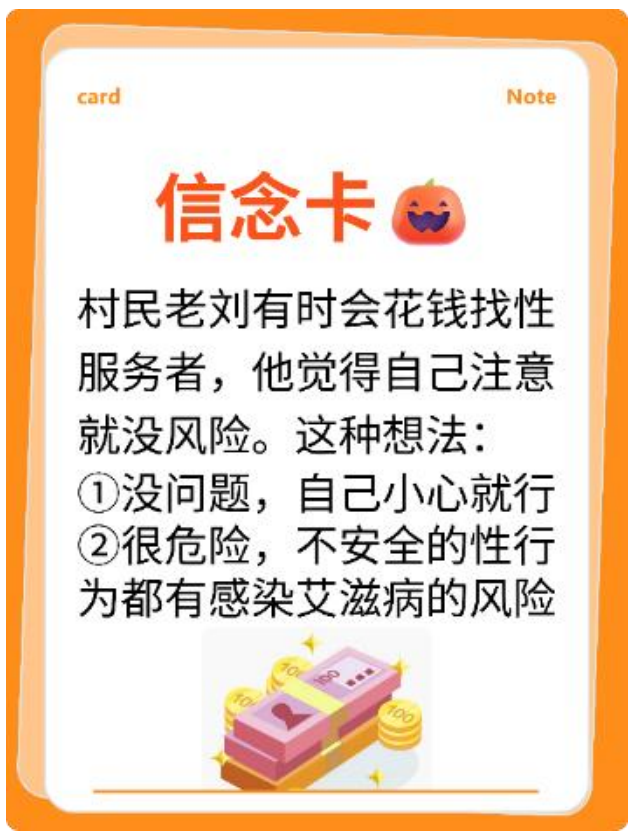	Attitude Card<br>Some villagers believe that paying for sex services carries no risk. This mindset:<br>① Is fine—just be careful<br>② Is dangerous—unsafe sex carries the risk of HIV infection	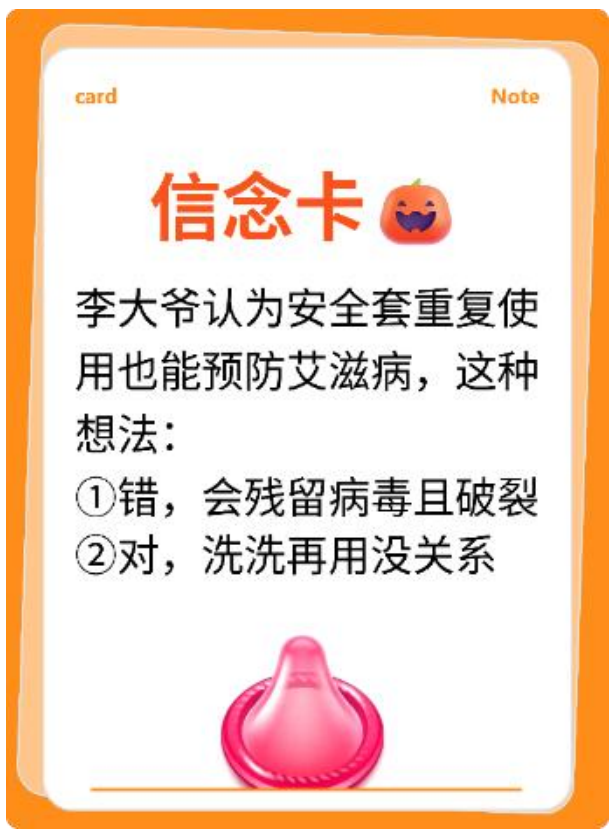	Attitude Card<br>Some villagers believe reusing condoms can prevent HIV infection. This belief:<br>① Correct: Washing and reusing is fine<br>② Incorrect: Viruses may remain and condoms may break
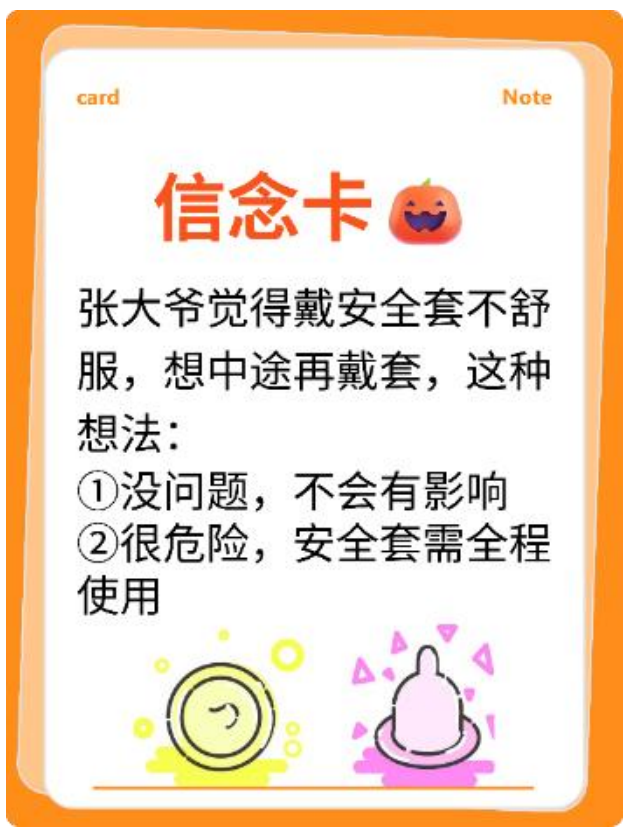	Attitude Card<br>Some villagers find condoms uncomfortable and want to put them on midway. Regarding this idea:<br>① It's fine and won't affect anything<br>② It's dangerous—condoms must be used throughout the entire act	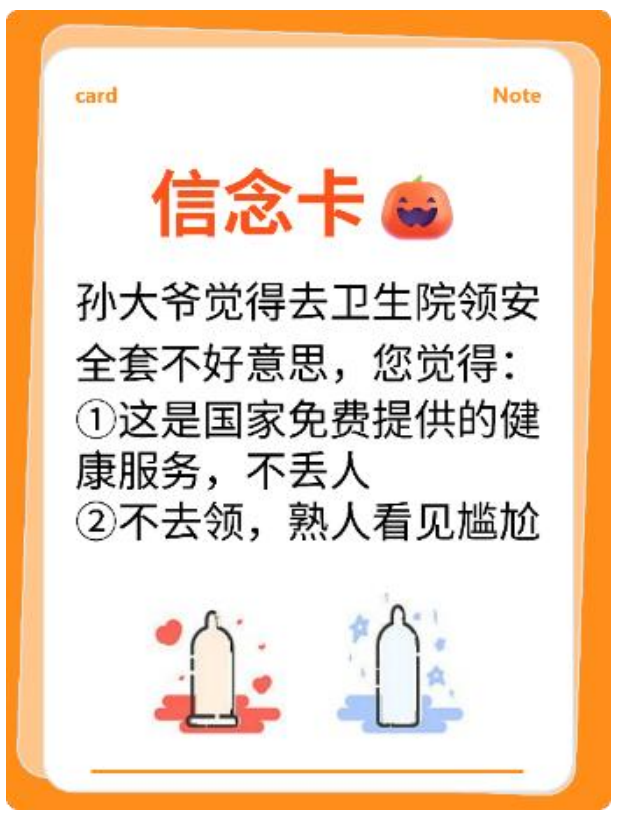	Attitude Card<br>Some villagers feel embarrassed about going to the health center to get condoms. What do you think?<br>① This is a free health service provided by the government—nothing to be ashamed of.<br>② Not going to get them would be awkward if acquaintances saw you.

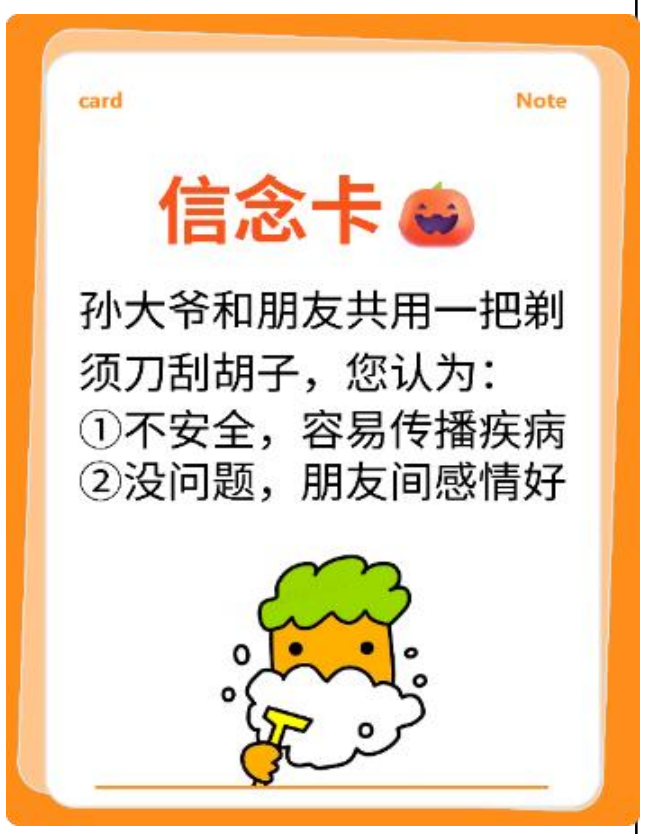	Attitude Card<br>Some villagers share a razor with friends for shaving. What do you think:<br>① Unsafe, prone to spreading diseases<br>② No problem, shows good friendship	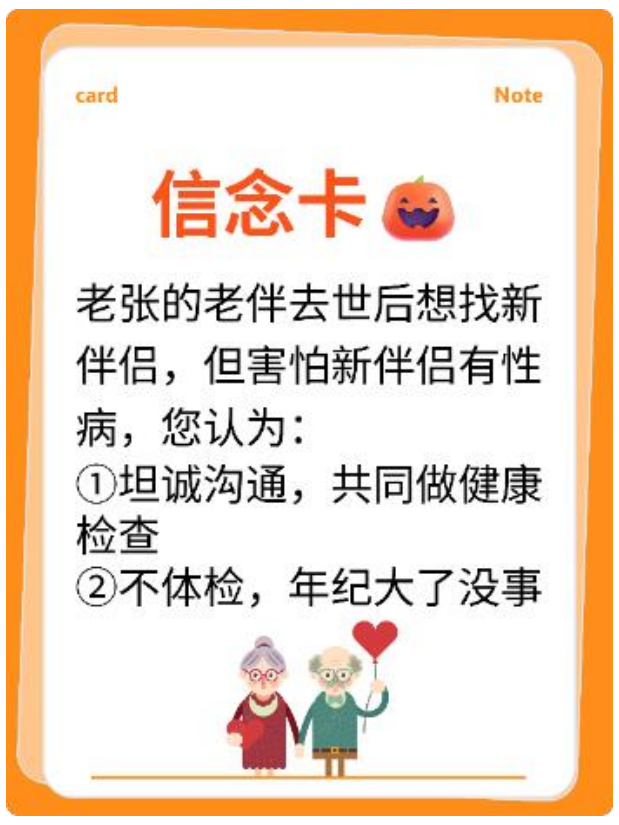	Attitude Card<br>A villager's boss passed away, and they now wish to find a new partner but fear the new partner might have an STD. What do you think:<br>① Be open and honest, get health checkups together<br>② Skip the checkup—it's okay since they're older
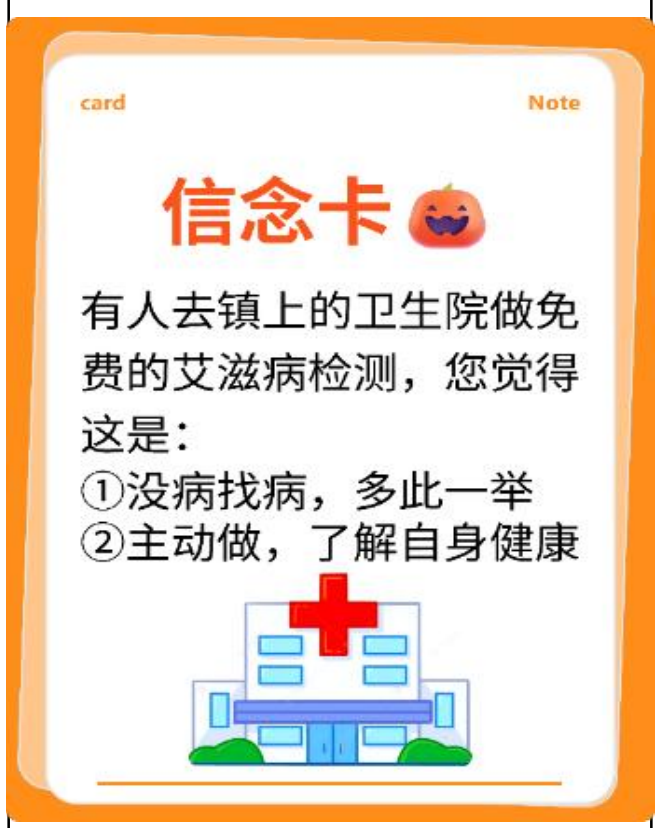	Attitude Card<br>Some villagers went to the town health center for free HIV testing. Do you think this is:<br>① Unnecessary, redundant<br>② Necessary, to understand one's own health	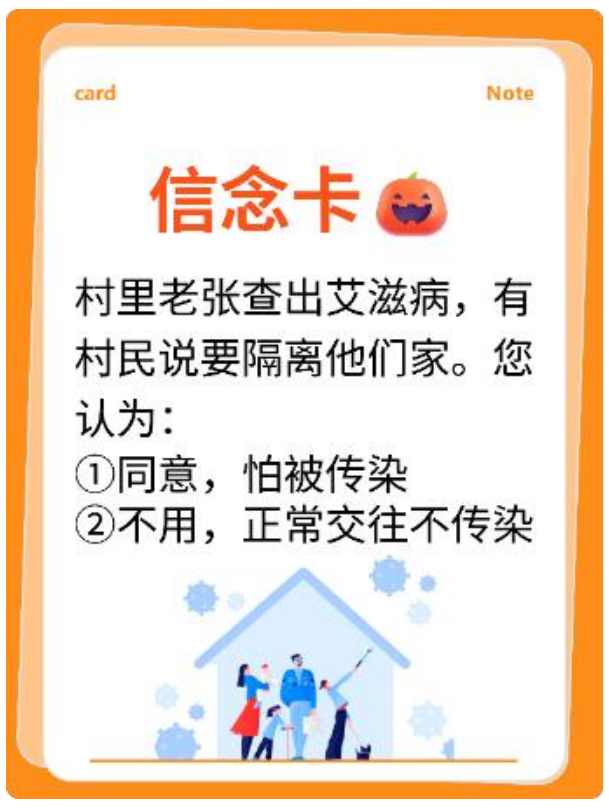	Attitude Card<br>Villager X has been diagnosed with HIV. Some villagers suggest isolating him. What is your stance?<br>① Agree, fearing infection<br>② Disagree, believing normal interaction won't transmit the disease
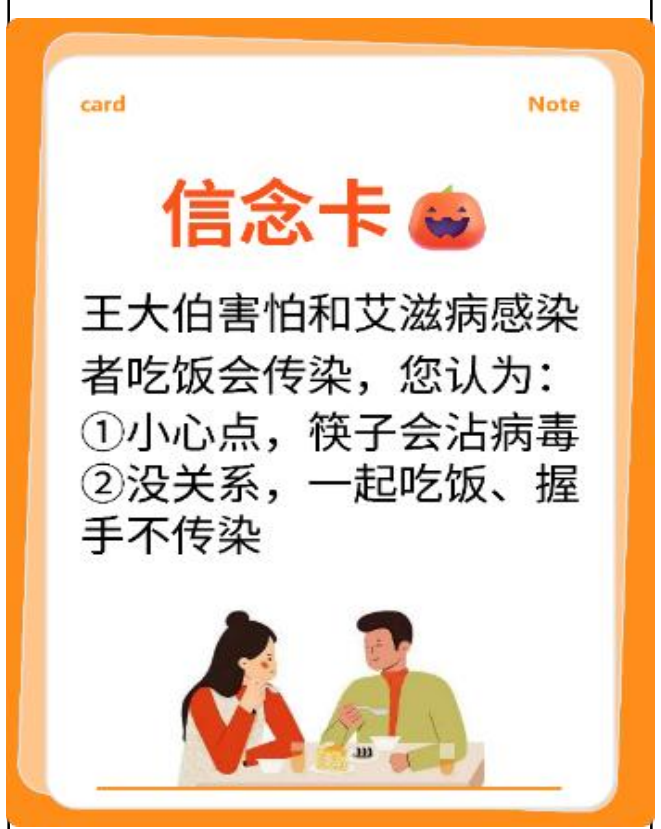	Attitude Card<br>Some villagers are afraid to eat with people living with HIV. Do you agree that:<br>① Yes, eating utensils can transmit the virus<br>② No, eating together does not spread the infection	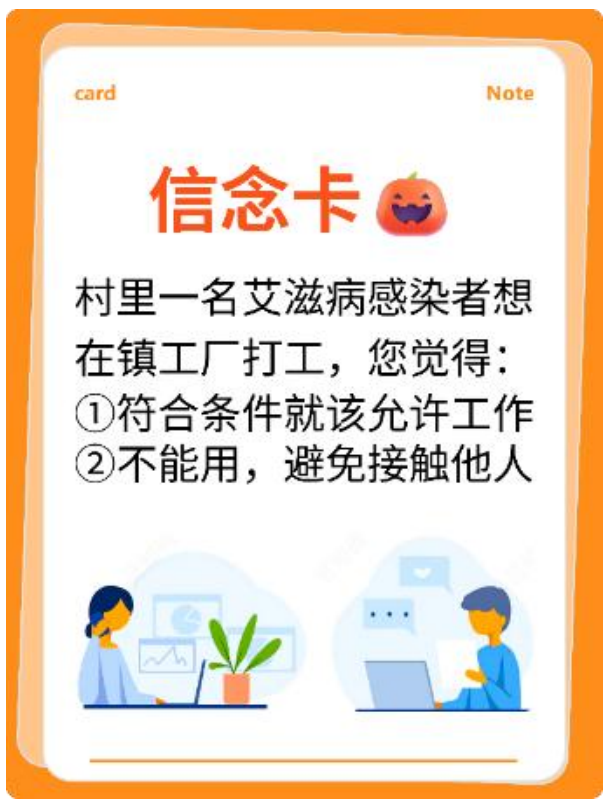	Attitude Card<br>A villager living with HIV wishes to work at a nearby factory. What is your stance?<br>① If they meet the requirements, they should be allowed to work.<br>② They should not be permitted to work to avoid contact with others.

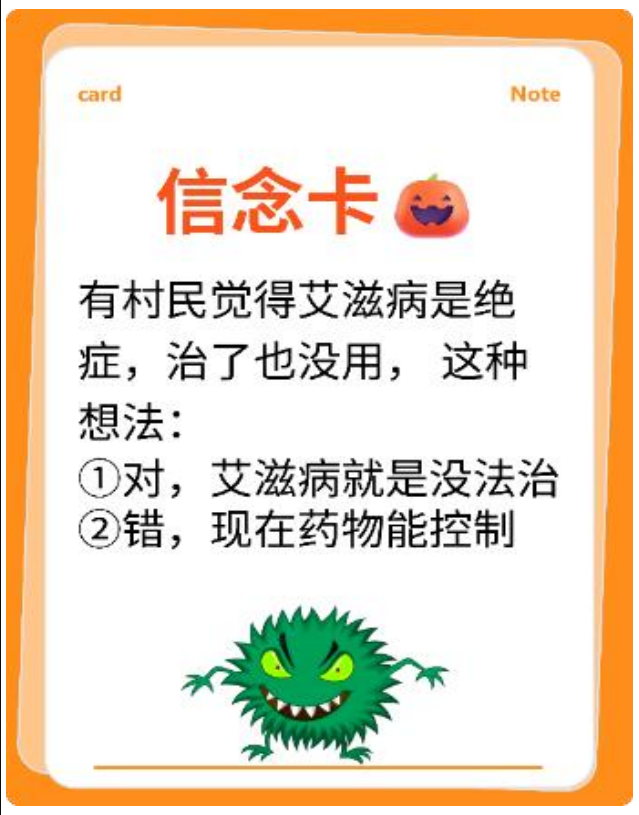	Attitude Card<br>Some villagers believe AIDS is a terminal illness that cannot be cured. This belief:<br>① Correct: AIDS patients can only wait to die<br>② Incorrect: Medication can control the progression of the disease	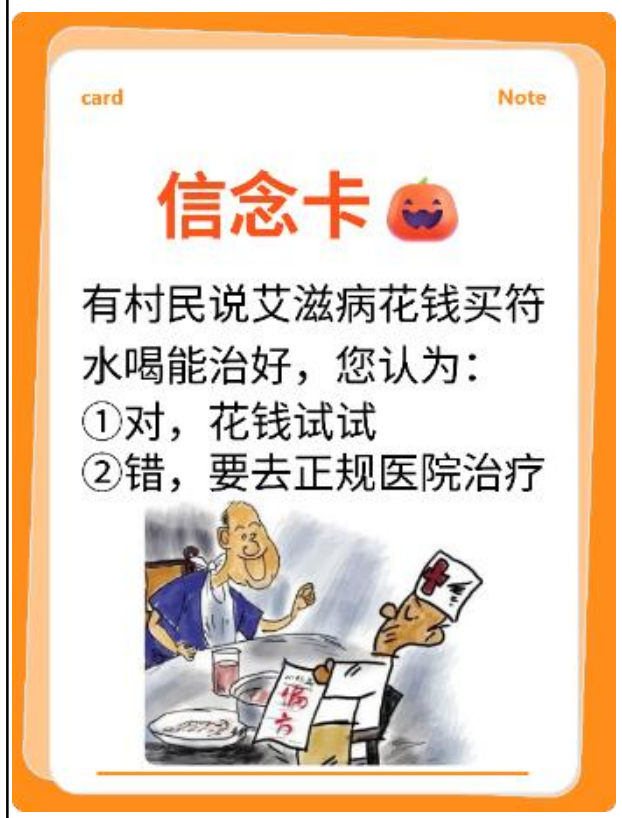	Attitude Card<br>Some villagers claim AIDS can be cured by spending money on folk remedies. Do you agree?<br>① Yes, spend more money and try it<br>② No, seek treatment at a proper hospital
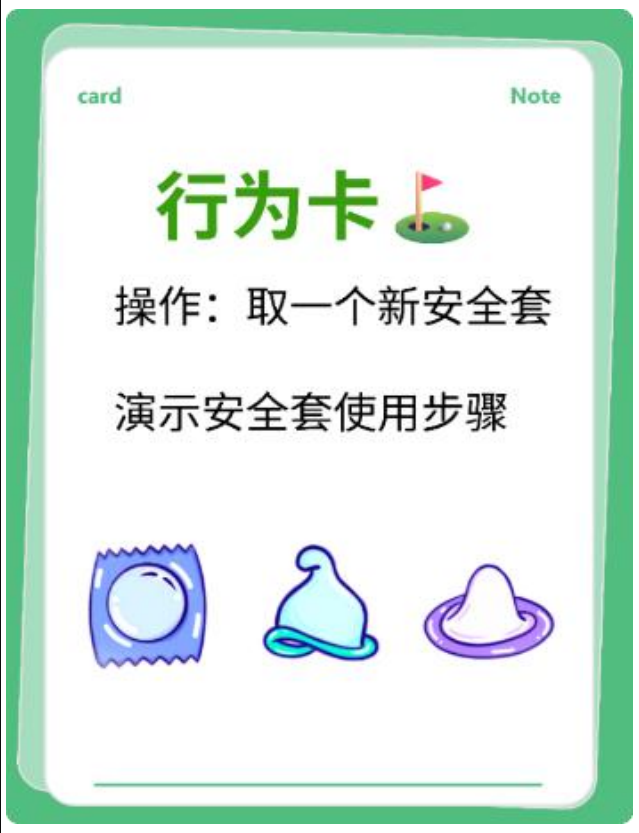	Behavior Card<br>Operation: Take a new condom and demonstrate the steps for putting it on.	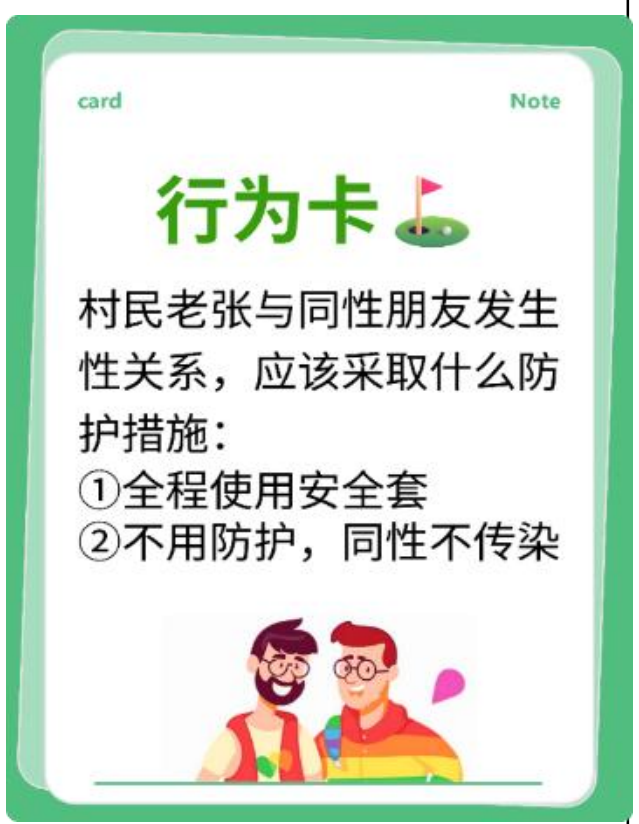	Action Card<br>Villager X has sex with a same-sex partner. What precautions should he take?<br>① Use condoms throughout the entire act<br>② No protection needed; same-sex partners don't transmit STDs
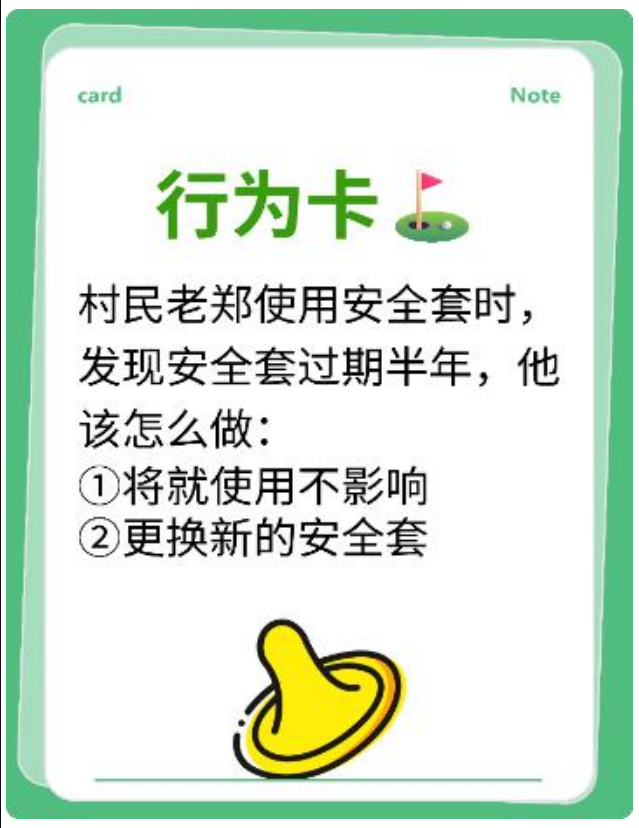	Behavior Card<br>Villager X discovers a condom has expired six months ago. What should he do?<br>① Make do with it—it won't affect anything<br>② Replace it with a new condom	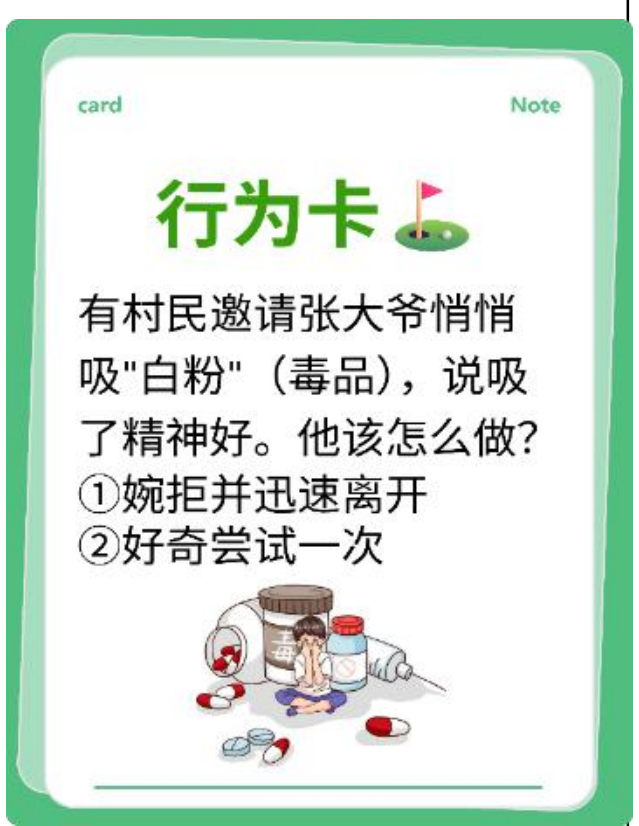	Behavior Card<br>A villager invites X to share a syringe for drug injection. What should he do?<br>① Try it out of curiosity once<br>② Politely decline and leave immediately

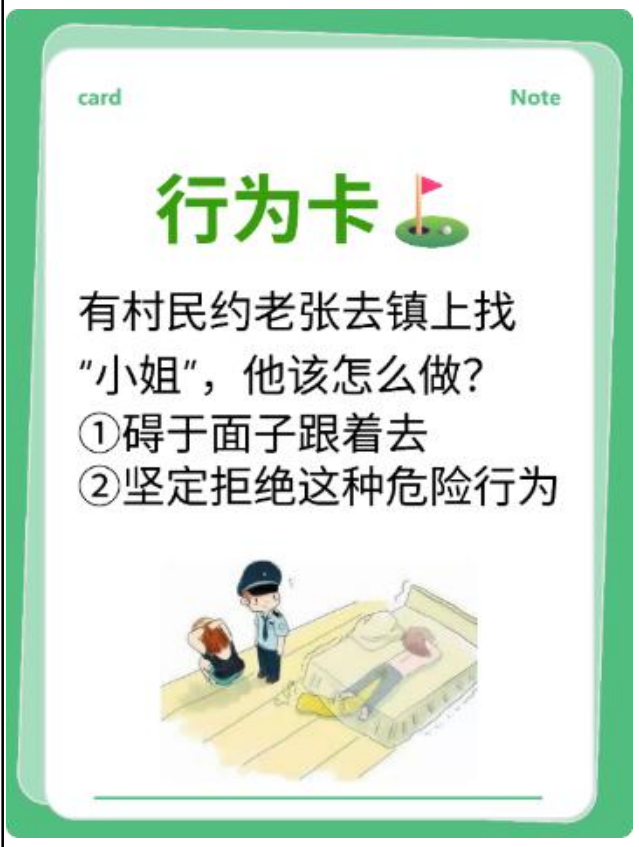	Behavior Card<br>A villager invites X to engage in commercial sex. What should he do?<br>① Go along without refusal<br>② Firmly refuse this high-risk behavior	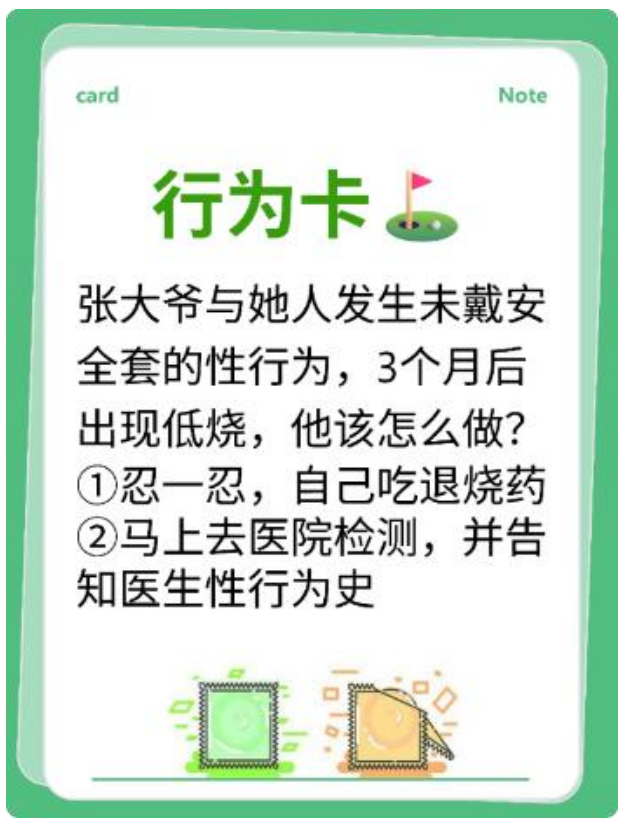	Behavior Card<br>X had unprotected sex with someone else and developed a low-grade fever three months later. What should he do?<br>① Tough it out and take over-the-counter fever reducers<br>② Go to the hospital immediately for testing and disclose his sexual history to the doctor
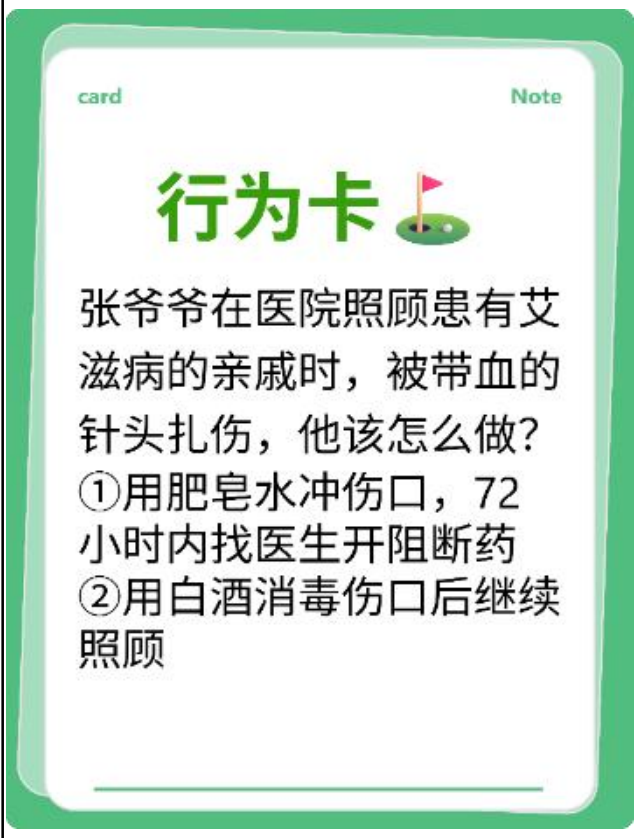	Action Card<br>X was caring for an HIV-positive relative in the hospital and was pricked by a needle with blood on it. What should he do?<br>① Disinfect with white liquor<br>② See a doctor within 72 hours to get post-exposure prophylaxis (PEP) medication	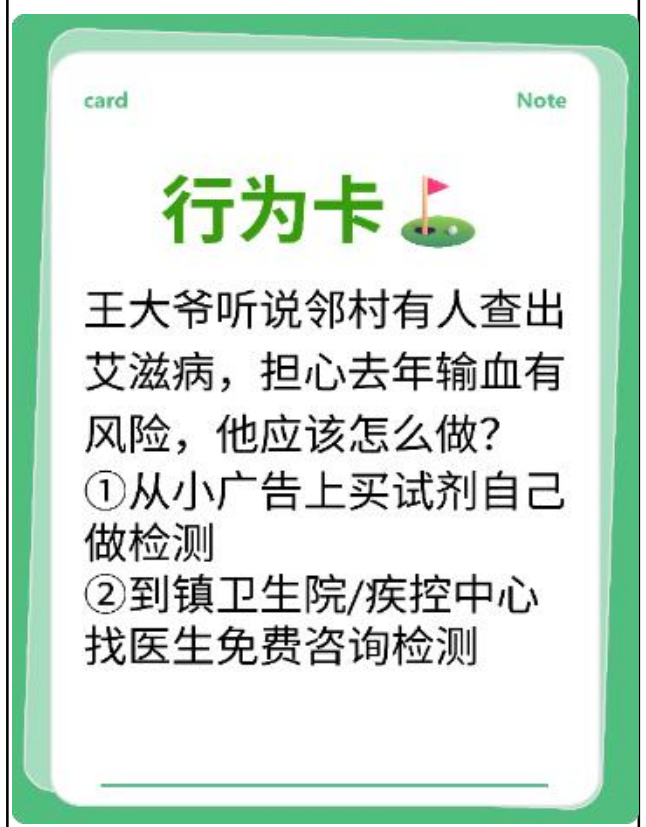	Action Card<br>X is worried about HIV infection and wants to get tested. What should he do?<br>① Purchase unregulated test kits for self-testing<br>② Visit the CDC for free consultation and testing with a doctor
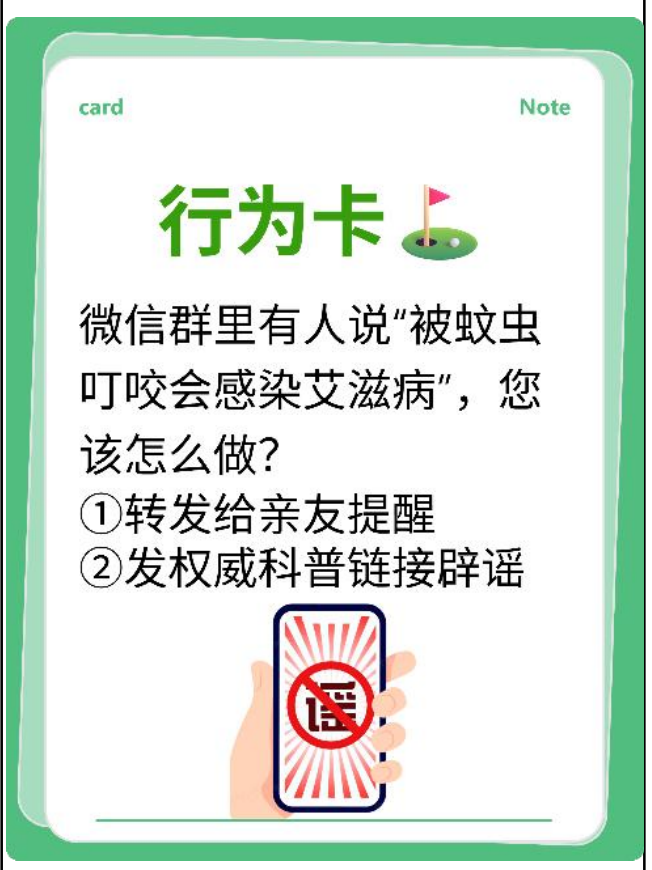	Behavior Card<br>Someone in a WeChat group claims “mosquito bites can transmit HIV.” What should you do?<br>① Forward this to friends and family as a reminder<br>② Share authoritative science-based debunking	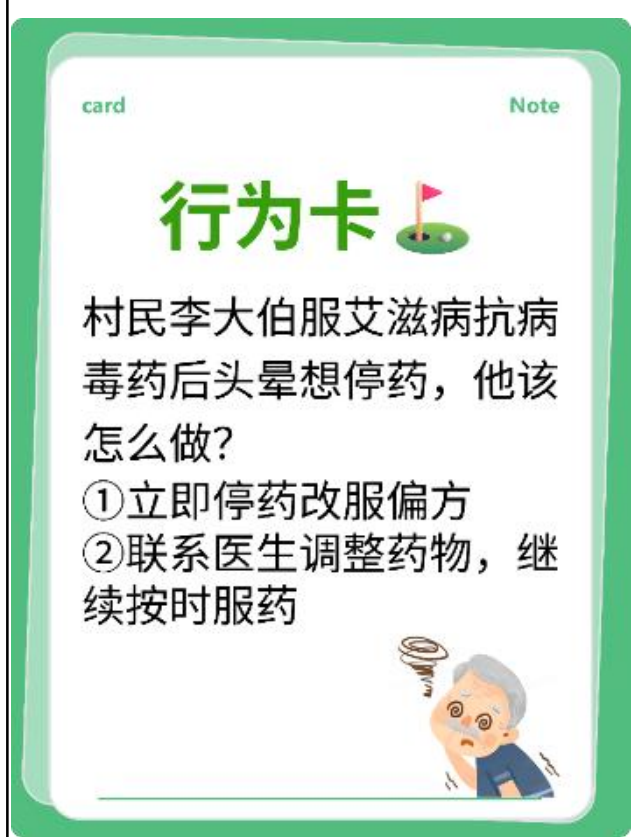	Behavior Card<br>Villager X feels dizzy after taking antiretroviral medication for HIV and wants to stop. What should he do?<br>① Immediately stop medication and switch to folk remedies<br>② Contact doctor for adjustment and continue taking medication on schedule
